# The Effectiveness of School-Based Interventions on Obesity-Related Behaviours in Primary School Children: A Systematic Review and Meta-Analysis of Randomised Controlled Trials

**DOI:** 10.3390/children8060489

**Published:** 2021-06-08

**Authors:** Sarah Nally, Angela Carlin, Nicole E. Blackburn, Judith S. Baird, Jo Salmon, Marie H. Murphy, Alison M. Gallagher

**Affiliations:** 1 Centre for Exercise Medicine, Physical Activity and Health, Sports and Exercise Sciences Research Institute, Jordanstown Campus, University of Ulster, Newtownabbey BT37 0QB, UK; nally-s@ulster.ac.uk (S.N.); a.carlin1@ulster.ac.uk (A.C.); mh.murphy@ulster.ac.uk (M.H.M.); 2 Centre for Health and Rehabilitation Technologies, Institute of Nursing and Health Research, School of Health Sciences, University of Ulster, Newtownabbey BT37 0QB, UK; ne.blackburn@ulster.ac.uk; 3 Nutrition Innovation Centre for Food and Health (NICHE), Biomedical Sciences Research Institute, Coleraine Campus, University of Ulster, Coleraine BT52 1SA, UK; baird-j7@ulster.ac.uk; 4 Institute for Physical Activity and Nutrition (IPAN), School of Exercise and Nutrition Sciences, Deakin University, Geelong 3217, Australia; jo.salmon@deakin.edu.au

**Keywords:** physical activity, sedentary behaviour, systematic review, meta-analysis, nutrition, school-based interventions, children

## Abstract

School-based interventions are promising for targeting a change in obesity-related behaviours in children. However, the efficacy of school-based interventions to prevent obesity remains unclear. This review examined the effectiveness of school-based interventions at changing obesity-related behaviours (increased physical activity, decreased sedentary behaviour and improved nutrition behaviour) and/or a change in BMI/BMI z-score. Following PRISMA guidelines, seven databases were systematically searched from 1 January 2009 to 31 December 2020. Two review authors independently screened studies for eligibility, completed data extraction and assessed the risk of bias of each of the included studies. Forty-eight studies met the inclusion criteria and were included in a narrative synthesis. Thirty-seven studies were eligible for inclusion in a meta-analysis. The findings demonstrate that interventions in children when compared to controls resulted in a small positive treatment effect in MVPA in the control group (2.14; 95% CI = 0.77, 3.50). There was no significant effect on sedentary behaviour, energy intake, and fruit and vegetable intake, and BMI kg/m^2^. A small significant reduction was found between groups in BMI z-score (−0.04; 95% CI = −0.07, −0.01) in favour of the intervention. The findings have important implications for future intervention research in terms of the effectiveness of intervention components and characteristics.

## 1. Introduction

Childhood obesity is one of the leading public health challenges of the 21st century [1]. The prevalence of childhood obesity has risen over the past four decades with 50 million girls and 74 million boys globally, aged 5–19 years, estimated to be affected by obesity [2]. Childhood obesity is a major economic concern and has serious health care and social costs and consequences in adulthood, with increased burdens on health systems [3]. The specific causes of obesity are varied and complex, however, at a population level are consistent with sustained positive energy balance [4,5].

Sedentary behaviour (SB) and low levels of physical activity (PA), combined with excess caloric consumption, are significant modifiable factors for the prevention of childhood obesity [6]. Childhood obesity is associated with adverse health outcomes, including greater adiposity, and associated adverse cardiometabolic risk factors [7,8], behavioural problems [9] and poorer academic performance [10]. Overconsumption and poor food choices are associated with a higher risk of developing childhood obesity [11,12,13]. Furthermore, dietary patterns during childhood that are high in energy-dense, high-fat and low-fibre foods will have an impact on the later obesity risk [14]. In addition to the well-recognised health benefits of PA, prolonged uninterrupted periods of sitting or SB are an independent risk for poor cardiovascular and metabolic health [15,16]. Increased sedentary time has been linked with changes in adiposity across childhood [17]. The 2018 Global Matrix PA Report Card, in 49 countries, indicated that only 27–33% of school-aged children were obtaining the recommended guidelines of 60 min of moderate-to-vigorous PA (MVPA) per day, and only 34–39% of those children adhered to the two hours of recommended screen time daily [18]. Therefore, evidence unequivocally highlights the beneficial effects of changes in PA, SB and nutrition behaviour for the prevention of childhood obesity [19,20,21,22]. When obesity is established in childhood, it is challenging to reverse through interventions and tracks into adulthood [23,24]. Thus, effective primary prevention is imperative. PA, SB, nutrition behaviour and food preferences are determined in early childhood and continue into adulthood [25,26,27]. By intervening at an early age, it may be possible to reduce health inequalities and prevent the increase in obesity levels [28].

Because of the prevalence, health consequences and associated costs of childhood obesity, there has been significant interest in identifying the effective interventions for preventing childhood obesity. Empirical evidence demonstrates that the school environment is ideal for tackling change in obesity-related behaviours as they provide concentrated contact, teach health education, provide meals and can model health-promoting settings [29]. It is a logical choice as a context for implementing obesity prevention interventions since children spend 40% of their waking time at school [30]. School-based interventions can also reach all pupils, irrespective of socioeconomic status [31,32] providing access to those who may benefit most and thereby overcoming potential health inequalities [33]. Multi-country data indicated that local school contexts have a significant impact on children’s daily MVPA accumulation pattern [34]. Recent systematic reviews and meta-analysis suggest that nutrition interventions can have a positive influence on children’s nutritional knowledge and children’s energy intake [35,36]. However, The National Institute of Clinical Excellence (NICE) has highlighted disparities in the evidence, specifically the need for evaluation of multi-component interventions that aim to reduce obesity-related behaviours in children [37].

Several reviews have considered the effectiveness of interventions promoting PA within the school setting [33,38] and during specific parts of a school day, including during play/recess [39], within-school physical education classes [40], and after-school [41,42]. The evidence is inconsistent and suggests that interventions promoting PA within-school, during play/recess and after-school may contribute to children’s PA accrual. Conversely, only a few systematic reviews have considered the effectiveness of SB interventions within the school setting [43,44]. A systematic review examining interventions for preventing obesity in children aged 6 to 12 years found no evidence to suggest that interventions that only focus on diet were effective [45]. Previous systematic reviews considering the effectiveness of school-based PA and dietary interventions have suggested that combined diet and PA interventions may help prevent childhood obesity [46,47]. However, these reviews of obesity prevention interventions in children and adolescents did not consider whether outcomes were influenced by methodological quality, characteristics of intervention components, intervention length and whether or not the interventions were theoretically driven. Further research is needed, to better understand how implementation of multi-component interventions deals with various intervention targets.

This review assesses the effectiveness of school-based interventions on primary school children at changing obesity-related behaviours and/or a change in BMI/BMI z-score, taking into account the methodological quality of the included studies. Secondary aims were to identify the most effective elements (e.g., PA, SB and/or nutrition), used in these interventions, to examine the characteristics of the interventions and to quantify their effect through meta-analysis.

## 2. Materials and Methods

This systematic review is reported in accordance with the PRISMA (Preferred Reporting Items for Systematic Reviews and Meta-Analyses) criteria [48] and was registered with PROSPERO (Registration number: CRD42019147930). The PRISMA checklist is provided in the Supplementary Materials (Table S1).

### 2.1. Search Strategy

A comprehensive search of seven electronic databases and online registers—Medline OVID (1946–2020), EMBASE (1980–2020), PsycINFO (1806–2020), SPORTDiscus (1984–2020), Scopus (1966–2020), CINAHL (1934–2020) and Cochrane Central Register of Controlled Trials (CENTRAL)—was conducted to identify peer-reviewed intervention studies published in English between 1 January 2009 and 25 December 2020. Searches were limited to that time period since a comprehensive review investigating the moderators of school-based intervention programmes on energy balance-related behaviours from inception to October 2009 was previously conducted by Yildirim et al. [49]. A research librarian (JA) was consulted during the development and testing of search terms. The search strategies combined multiple keyword search terms agreed to a priori and were developed by breaking down the research question. The search terms focused on four key elements: (1) study population; (2) outcome measure; (3) study type; and (4) setting. As an illustration, the search strategy used in Medline is included in the Supplementary Information (Table S2). Reference lists of included studies were hand-searched for eligible interventions. The outcomes of each of the searches were combined into a RefWorks library (bibliographic software).

### 2.2. Inclusion and Exclusion Criteria

Studies were eligible if they targeted children aged 5 to 12 years of any nationality, attending primary school on a full-time basis. If the ages of participants fell outside of this range, the mean age had to be within this range to warrant inclusion. Participants were only included if they were under 13 years at the commencement of the intervention. Studies were also eligible if they: (1) included an intervention that lasted at least 12 weeks; (2) were written in the English language and published after 2009; (3) targeted a change in at least two measures of BMI and/or obesity-related behaviours including PA behaviour and/or SB and/or nutrition behaviour; (4) randomised controlled trials (RCTs) or cluster-randomised controlled trials (cRCTs) with a comparison or control arm that consisted of either no intervention, an alternative treatment condition or ‘usual care’, i.e., existing physical education programme.

Studies were excluded if they: (1) included children with medical conditions known to affect weight status, children with eating disorders, critical illness, asthma or other chronic conditions or children with mental or physical disabilities; (2) were based primarily outside of the school setting (e.g., community settings, public place, at home, recreation facility, hospital, camp setting) or in a clinical population; and (3) involved participants receiving a PA intervention as part of a treatment regimen for a specific critical illness or comorbidity.

### 2.3. Study Selection

All search results were exported into the RefWorks library, and duplicates were removed. Title and abstract of retrieved articles were screened independently by two authors (SN and JB), using the inclusion/exclusion criteria as described above. When full texts were not readily available, the lead author was contacted and requested to provide the full text for further assessment on eligibility. If no response was received, these studies were excluded as they could not be fully assessed for eligibility. If a study was mentioned multiple times, only the most recent publication was included in the analysis. Reference lists of the included studies and related systematic reviews were examined to identify any additional studies. Two authors (SN and JB) then independently reviewed the full text of the remaining articles to determine final inclusion. When uncertainties arose with regard to the study inclusion, consensus was achieved through discussion with a third author (AG). The third author was consulted for five articles.

### 2.4. Data Extraction Process

Data extraction was conducted in the selected studies by the first author (SN), and a second reviewer (AC) double-checked the data extraction of all the included studies. Study characteristics, intervention characteristics, description of the intervention using the Template for Intervention Description and Replication (TIDieR checklist) [50] and effectiveness of the interventions were all extracted. The characteristics of interventions were extracted from both the main papers and where relevant from the published intervention protocol including: sample size, age of participants, study design, number of schools participating in the study, description of intervention, intervention type, timing and frequency of activities, providers, setting, resources and equipment used, underlying behavioural theory, training, the timeframe in which follow-up measurements were made, drop-out rates, characteristics of the participants, outcome measures and details of the control group.

Information was extracted on whether the single-component interventions targeted a change in PA, SB or nutrition behaviour. Interventions covering multiple components that targeted a change in two or more obesity-related behaviours were also extracted. The TIDieR checklist was included in data extraction following recent guidance for improving systematic reviews [51].

### 2.5. Evaluation of the Quality of the Studies

Two authors (SN and AG) independently assessed the risk of bias of all included trials using the Cochrane risk of bias tool [52]. Studies were assessed based on selection bias (i.e., random sequence generation, allocation concealment), detection bias (i.e., blinding of study personnel), attrition bias (high is less than 70% at follow-up) and the validity of the outcome measure included in the intervention (i.e., device-based versus self-reported measures). A judgement of ‘low risk’, ‘high risk’, or ‘unclear risk’ of bias was selected for each of the domains. Disagreements were resolved through discussion until consensus was reached. If no consensus was reached, a third author independently assessed the risk of bias for that domain.

### 2.6. Meta-Analysis

The effect sizes were determined (mean differences) reporting 95% CI for the difference between arms (intervention vs. control) for each of the three behavioural outcomes and for BMI, as implemented into Review Manager (RevMan) (*Version 5.3. Copenhagen: The Nordic Cochrane Centre, The Cochrane Collaboration, 2014*). Data were pooled to compare the post intervention mean differences between intervention vs control for each of the outcomes. For data presented in the form of odds ratios or counts, a meta-analysis was conducted using the generic inverse variance method as implemented by RevMan. The difference between the intervention groups and control/comparison group in the mean change from baseline to post-intervention and the comparison at follow-up were used as a measure of effect size. Where study authors reported both device-measured and self-reported measurements for PA and/or SB, only the device-based measurement data were used in the statistical analysis. We quantified the extent of the variability observed that could be accounted for by true between-study differences rather than chance using the heterogeneity I^2^ statistic [53]. Results with *p* < 0.05 were reported as significant.

Pre-planned subgroup analyses were explored on possible moderators of the average intervention effect, and these were: PA and SB measurement method (device-based vs. self-report), intervention duration (<6 months vs. >6 months) and presence of underpinning theory (yes vs. no). Additional pre-planned subgroup analyses explored treatment-subgroup interactions with the type of intervention (multiple components or single component). Subgroup analyses, although planned, were exploratory; adjustments for multiple comparisons were applied.

Sensitivity analyses were performed for random-effects meta-analyses by strategically removing studies one by one if they had a small sample size, high risk of bias, sedentary time or PA measures that were parent-reported compared to device measured to assess the robustness of the summary estimates. This would also indicate whether an individual study accounted for a large proportion of the heterogeneity. I^2^ is represented as a percentage with a value of 0% indicating no dispersion and larger values indicating gradual increases in heterogeneity (i.e., 25% = low, 50% = moderate, 75% = high level of heterogeneity) [53]. To identify any potential outliers, a set of leave-one-out analysis was performed to identify potential significant studies that resulted in a large change in the pooled estimates after they were left out one at a time from all studies.

Publication bias was assessed by examining asymmetry of funnel plots (effect size vs. standard error) where asymmetry is indicative of publication bias. We recognised that asymmetry of funnel plots can be due to either publication bias or a genuine relationship between effect size and trial size. To assess the possibility of publication bias, a minimum of 10 studies for a given outcome was required for the meaningful interpretation of funnel plots [54,55].

## 3. Results

### 3.1. Database Searches

**Figure 1:**
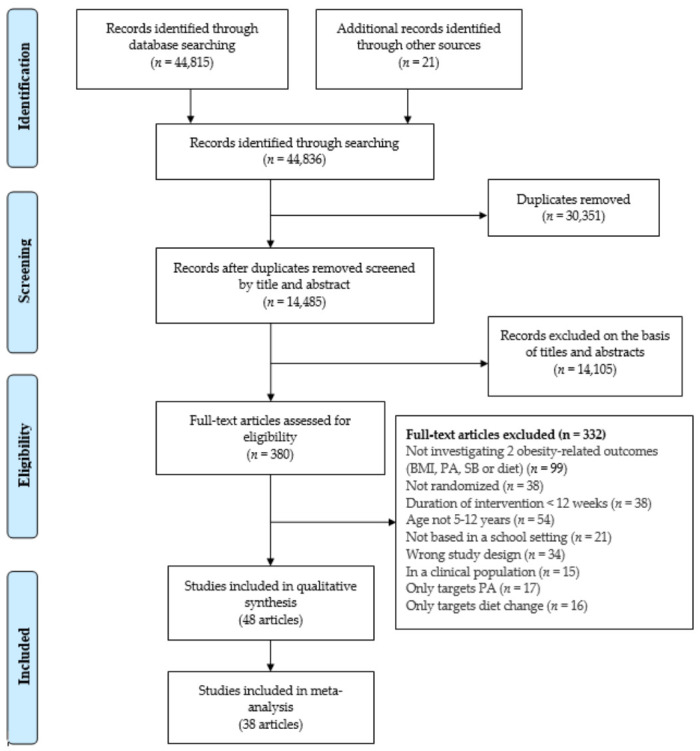
Flowchart of study selection.

A PRISMA flowchart in Figure 1 illustrates the identification, screening, eligibility and inclusion of studies within the systematic review. The database search yielded 44,815 articles, while 21 additional articles were retrieved after reviewing the reference lists of the included studies. A total of 48 articles met the inclusion criteria for the narrative synthesis. Thirty-eight studies were eligible for inclusion in a meta-analysis.

### 3.2. Description of the Included Studies

The characteristics of the included studies are described in the Supplementary Information (Table S3). Most of the studies were cRCTs (*n* = 35, 73%) using the school or class as the unit of randomisation. The remaining studies were RCTs (*n* = 13, 27%). The 48 articles within this review involved a total of 46,235 children at baseline. The number of children participating in the study at baseline ranged from 51 [56] to 3135 [57].The average age of participants in each study ranged from 6.0 years [58] to 10.9 years [59,60]. Whilst the participant groups were quite similar in terms of age across studies, the interventions were heterogeneous regarding study duration, intensity, type of the interventions and outcome measures. The duration of the interventions ranged from 12 weeks [61] to four years [62], 19% of interventions (*n* = 9) had a duration of less than six months and 81% of the interventions were over six months in duration (*n* = 39). The number of schools participating in the studies ranged from one school [56] to 154 schools [63,64]. In this review, interventions were conducted within twenty different countries, including ten European countries (*n* = 25, 52%), within the USA (*n* = 8, 17%), Asia (*n* = 7, 15%) and in two Oceania countries (*n* = 4, 8%), and the remaining four studies were conducted in Argentina, Canada, Chile and Mexico (Table S3). Of the 48 articles included in the qualitative synthesis, 43 (90%) articles targeted a change in PA, 29 (64%) articles targeted a decrease in SB, and 30 (63%) articles targeted change in nutrition behaviour (Table 1). Seven different methods were used to measure PA and SB. Both PA and SB were objectively measured via accelerometer in 13 studies [65,66,67,68,69,70,71,72,73,74] and subjectively measured through self-report questionnaires in 12 studies [58,60,69,75,76,77,78,79]. One study used a combination of self-report and accelerometers to measure PA and SB [80], and three studies used questionnaires to measure both PA and SB [63,64,81]. Two studies used three different methodologies to measure PA and SB including direct observation and device-based measurements [62,82]. Seven studies measured PA using an accelerometer [57,61,83,84,85,86], and four studies measured PA subjectively via questionnaires [87,88,89,90]. To measure PA, one study combined self-report and accelerometers [91], another study combined pedometers and self-report measurements [56], one used pedometer [92], and one used a 7-day PA recall [59].

A variety of dietary assessments were employed throughout the 30 articles. Food frequency questionnaires were the most commonly used [57,58,65,66,69,76,77,81,93,94,95,96]. One study used the digital photography method on three consecutive days to measure food selections and food intake [79]. Viggiano et al. [90], used a graphical form of a food diary with a list of items to evaluate the quantity and quality of food consumption during of 7-day period. Two studies used a 24-h recall food intake questionnaire [68,87], two studies used a 3-day dietary recall sheet, on two weekdays and one day on the weekend, to estimate dietary intake [60,97], and one study used the 24-h food recall method among a subset of participants on a day that was convenient for the participants (*n* = 135) [98]. Two studies measured children’s nutrition behaviours via a parental questionnaire [63,64,99]. The mode of nutritional intervention delivery varied; however, the most used intervention strategy was health education classes and nutrition education programmes. One study used the board game ‘Kaledo’ as a strategy to improve nutrition knowledge to modify nutrition behaviour [90].

There were 35 (71%) multi-component interventions, while others adopted single component and 21 (48%) multi-component interventions targeted a change in PA, SB and nutrition behaviour [56,58,60,63,64,65,68,69,70,76,79,80,81,82,91,95,100,101]. Eight (17%) interventions targeted a change in PA and SB [62,67,71,72,73,74,75,81], and five (9%) interventions targeted a change in PA and nutrition behaviour [57,77,86,87,90]. Intervention strategies included school environment adaptions, interactive drama activities, modified PE lessons, extra-curricular PA sessions, gardening, cooking workshops, educational sessions, counselling sessions and provision of further opportunities to be physically active (e.g., active homework, lunch and break time, PA clubs). Fourteen (30%) studies reported single-component interventions. Eight of these were targeting a change in PA [59,61,83,84,85,89,92,97] by facilitating active academic lessons, activity breaks in the classroom, introducing additional brisk walking during school time, school environment adaptions and educational sessions. Six single-component interventions targeted a change in nutrition through workshops and modifications to the school canteen [94], allocating free fruit [102] and educational sessions [88,93,96,98].

**Table 1:** Characteristics of included interventions targeting a change in obesity-related behaviours and/or body mass index (*n* = 48).

Study, Year	Outcomes				PA		SB		Theory Based	Duration (>6 Months)
	PA	SB	NB	BMI	Device-Based	Self-Report	Device-Based	Self-Report		
Adab et al., 2018 [65]	✓	✓	✓	✓	✓		✓			✓
Amini et al., 2016 [60]	✓	✓	✓	✓		✓		✓		
Anderson et al., 2016 [66]	✓	✓	✓	✓	✓		✓		✓	✓
Angelopoulos et al., 2009 [87]	✓		✓	✓		✓			✓	
Bacardí-Gascon et al., 2012 [80]	✓	✓	✓	✓	✓			✓	✓	
Bere et al., 2014 [102]			✓	✓						✓
Brandstetter et al., 2012 [99]	✓	✓	✓	✓		✓		✓	✓	✓
Donnelly et al., 2009 [83]	✓			✓	✓					✓
Drummy et al., 2016 [61]	✓			✓	✓					
Efstathiou et al., 2016 [81]	✓	✓	✓			✓		✓		✓
Engelen et al., 2013 [67]	✓	✓		✓	✓		✓			
Fairclough et al., 2013 [68]	✓	✓	✓	✓	✓		✓		✓	
Farmer et al., 2017 [84]	✓			✓	✓					✓
Ford et al., 2013 [97]	✓		✓	✓	✓					
Habib-Mourad et al., 2020 [77]	✓		✓			✓			✓	✓
Howe et al., 2011 [59]	✓			✓	✓					✓
Kain et al., 2014 [92]	✓			✓	✓					✓
Khan et al., 2014 [98]			✓	✓						✓
Kobel et al., 2014 [63]	✓	✓	✓	✓		✓		✓	✓	✓
Kobel et al., 2017 [64]	✓	✓	✓	✓		✓		✓	✓	✓
Kocken et al., 2016 [91]	✓	✓	✓	✓	✓			✓	✓	✓
Lau et al., 2016 [85]	✓			✓	✓					
Li et al., 2019 [69]	✓	✓	✓	✓	✓		✓		✓	✓
Liu et al., 2019 [101]	✓	✓	✓	✓		✓		✓	✓	✓
Llargues et al., 2011 [58]	✓	✓	✓	✓		✓		✓		✓
Llaurado et al., 2014 [103]	✓	✓	✓	✓		✓		✓		✓
Lloyd et al., 2018 [100]	✓	✓	✓	✓	✓		✓		✓	✓
Lynch et al., 2016 [56]	✓	✓	✓	✓	✓			✓		
Madsen et al., 2015 [70]	✓	✓	✓	✓	✓		✓			✓
Marcus et al., 2009 [57]	✓		✓	✓	✓					✓
Martinez-Vizcaino et al., 2014 [71]	✓	✓		✓	✓		✓		✓	✓
Nathan et al., 2020 [72]	✓	✓			✓		✓		✓	✓
Nickel et al., 2020 [96]			✓	✓						✓
O’Leary et al., 2019 [86]	✓			✓	✓				✓	✓
Rausch Herscovici et al., 2013 [94]			✓	✓						✓
Resaland et al., 2016 [73]	✓	✓		✓	✓		✓		✓	✓
Rosario et al., 2017 [88]	✓			✓		✓			✓	
Sacchetti et al., 2013 [75]	✓	✓		✓		✓		✓		✓
Santina et al., 2020 [78]	✓	✓		✓		✓		✓	✓	
Scherr et al., 2017 [93]			✓	✓					✓	✓
Seljebotn et al., 2019 [74]	✓	✓		✓	✓		✓			✓
Siegrist et al., 2013 [89]	✓			✓		✓				✓
Tarro et al., 2014 [76]	✓	✓	✓	✓		✓		✓		✓
Telford et al., 2016 [62]	✓	✓		✓	✓		✓			✓
Viggiano et al., 2018 [90]	✓		✓	✓		✓				
Wells et al., 2014 [82]	✓	✓			✓		✓			✓
Williamson et al., 2012 [79]	✓	✓	✓	✓		✓		✓	✓	✓
Xu et al., 2015 [95]	✓		✓	✓		✓		✓	✓	✓

Abbreviations: PA = physical activity, SB = sedentary behaviour, NB = nutrition behaviour.

One study used several intervention activities including: classroom lessons, 19 take-home activities, cooking demonstrations, school gardens, family newsletters, health fairs, salad bars, procurement of regional produce and school-site wellness committees [93]. Four interventions did not measure BMI at any time point [72,77,81,82]. One study used a self-reported measurement for height and weight [103]; the remainder of the interventions measured the participants’ body weight and height objectively by a member of the research team.

Twenty-one (44%) studies explicitly reported that the interventions incorporated one or more behaviour change theories (Table 1). These included Social Cognitive Theory [63,64,66,68,77,88,93,99,100], Social Identity Theory [78], the Theory of Planned Behaviour [87,91], Socio-Ecological Model [71,73,93], RE-AIM (Reach, Efficacy, Adoption, Implementation, Maintenance) Theoretical Framework [72,86], the Theory of Triadic Influence and the Comprehensive School Health Program Model [79], Bronfenbrenner’s Ecological Model [77] and the analysis grid for environments linked to obesity (ANGELO) [101]; two studies used a theoretical pathway that was informed by a previous mixed-method study [58,62,65,69]. The remaining studies (*n* = 27, 56%) did not specify the use of a behaviour change model or theory.

### 3.3. Quality of Included Studies

**Figure 2:**
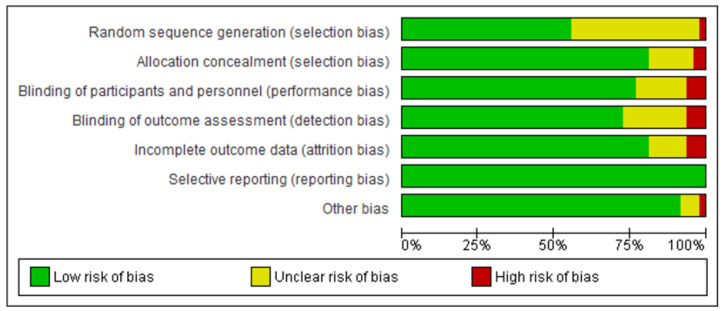
Summary of assessment of risk of bias (*n* = 48).

Assessment of risk of bias is summarised in Figure 2. All the studies in this review had a low risk of bias for selective reporting (*n* = 48, 100%). Approximately half of the studies were assessed as having an unclear risk of bias due to insufficient descriptions in terms of random sequence generation (*n* = 21, 44%). Most of the interventions were judged as having a low risk of bias in terms of selection bias (*n* = 38, 79%), performance bias (*n* = 37, 77%), detection bias (*n* = 34, 71%) and attrition bias (*n* = 39, 81%). The studies that were judged as having the highest risk of bias were for incomplete outcome data, blinding of outcome assessment and performance bias.

### 3.4. Meta-Analysis

Of the 48 studies included in the qualitative synthesis, 37 studies provided sufficient data for inclusion in the meta-analysis. Studies were excluded for the following reasons: not reporting sample size, missing data, inappropriate data type (i.e., studies providing different measures of variation, e.g., range, reference ranges and studies stating ‘not significant’ or ‘*p* < 0.05’) and not reporting variance of data.

### 3.5. Effects of Interventions on Changing Moderate-to-Vigorous Physical Activity

Of the 37 studies included in the meta-analysis, 18 studies provided data for MVPA. For studies in which two measures of MVPA were obtained, data were extracted for the device-based measurement only. Assessment of the effect of interventions on MVPA comprised of 9263 participants indicating a small but statistically significant increase in MVPA in favour of the control group (2.14; 95% CI = 0.77, 3.50) with moderate inconsistency across trials (I^2^ = 66%). Subgroup analyses were performed as planned to explore whether the identified subgroups moderated the average intervention effect. We found several significant treatment subgroup interactions that could explain this inconsistency (Table 2).

### 3.6. Effects of Interventions on Changing Sedentary Behaviour

Of the 37 studies included in the meta-analysis, 16 studies provided sufficient data for SB. Twelve studies provided adequate data for objectively assessed sedentary time (mins/day) for which the pooled ES estimates were −0.91 (95% CI = −2.30, 0.48). This estimate did not reach statistical significance, with low precision, as indicated by the CIs from a negative large effect to a positive large effect. Several significant treatment subgroup interactions were noted in Table 2. There were greater treatment effects when treatment duration was more than six months and when the measurement was device-based. Anderson et al. [66] measuring screen time (mins/day) (10.17; 95% CI = −1.58, 21.92), Santina et al. [78] (13.80; 95% CI = −0.96, 28.56) and an alternative study by Amini et al. [60] measuring TV viewing (mins/day) (−3.00; 95% CI = −4.44, −1.56) found no significant effect, and all had very low precision.

### 3.7. Effects of Interventions on Changing Nutrition Behaviour

Eleven studies that aimed to enhance healthy dietary behaviour were included in the meta-analysis. Four RCTs including 1576 participants showed a non-significant increase in energy intake (kcal/day) (5.23; 95% CI = −77.83, 88.28). A separate meta-analysis was conducted in five studies (*n* = 4741) to investigate intervention effects on portions of fruit and vegetables consumed per day (0.05; 95% CI = −0.08, 0.17), and two studies examined fruit and vegetable intake (g/day) (10.45; 95% CI = −17.53, 38.43); all showed a non-significant increase in fruit and vegetable consumption. Although heterogeneity from the pooled analysis was low (I^2^ = 0%), the individual effects from the included studies were extremely inconsistent. All other planned subgroup analyses were non-contributory.

### 3.8. Effect of Interventions on Changing BMI and BMI z-Score

**Figure 3:**
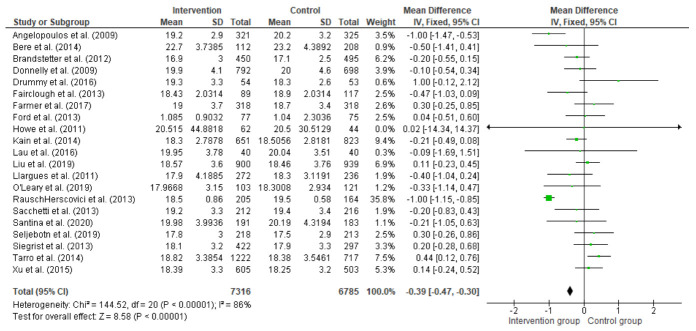
Difference between intervention and control groups of school-based interventions in primary school children at follow-up in BMI (kg/m^2^).

**Figure 4:**
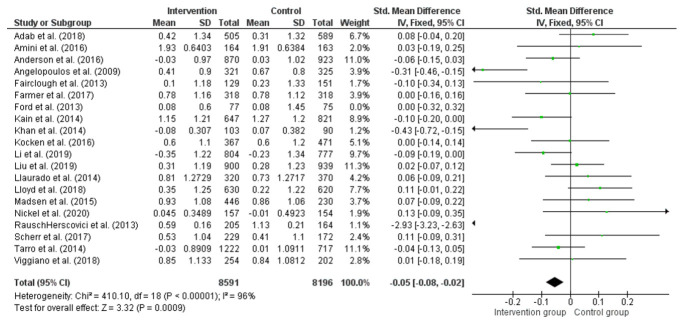
Difference between intervention and control groups of school-based interventions in primary school children in BMI z-score.

The overall effect size in 20 studies for BMI kg/m^2^ is summarised in Figure 3, and the overall effect size in 16 studies for BMI z-score is summarised in Figure 4. The quantitative synthesis of the interventions showed a non-significant reduction in BMI (−0.04 kg/m^2^; 95% CI =−0.13, 0.05) and a small significant reduction in BMI z-score (−0.04; 95% CI = −0.07, −0.01) compared with the control group. Sensitivity analysis was conducted in studies measuring change from baseline rather than follow-up data in BMI (kg/m^2^), removal of Ford et al. [97] = (−0.04; 95% CI = −0.13, 0.05). Subgroup analyses indicated that means of BMI differed significantly by whether they were theory-based and interventions of more than six months in duration (Table 2).

### 3.9. Assessment of Publication Bias

**Figure 5:**
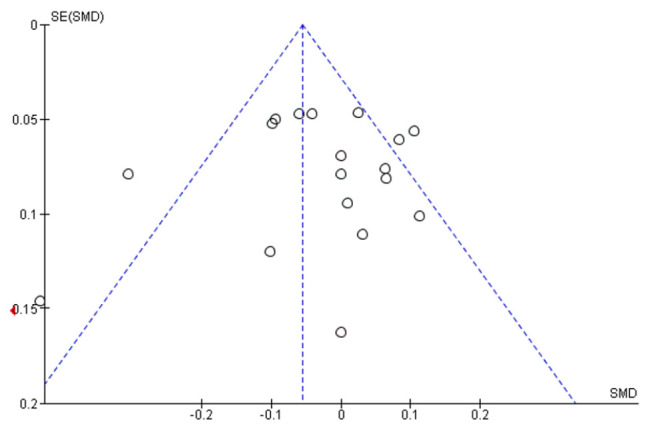
Assessment of publication bias: funnel plot (BMI z score).

A subjective evaluation of the funnel plot showed a slightly asymmetric scatter consistent with publication bias (Figure 5).

## 4. Discussion

This review and meta-analysis synthesised the evidence of the efficacy of school-based interventions at changing obesity-related behaviours among primary-school children. Meta-analysis results indicate that school-based interventions had a small significant effect in BMI z-score compared with controls, but no significant effect on sedentary time, energy intake, fruit and vegetable intake and BMI (kg/m^2^). Subgroup analyses found studies lasting more than six months targeting a change in MVPA were more effective than studies that were of a shorter duration. In addition, we found significant interaction between theory-based interventions and interventions lasting more than six months and the effectiveness of changing BMI.

In this review, the overall effects in terms of BMI/BMI z-score and weight reductions were in favour of the intervention group, and these findings align with previous reviews of interventions that target childhood obesity [104,105]. Nevertheless, the sustainability of any reduction detected in BMI/BMI z-score is an important factor, and there is a need for a long-term follow-up [106]. Comparison of review findings must be examined with caution due to the varying eligibility criteria such as age of participants, study design and setting. Moreover, BMI as an outcome of these interventions may be relatively insensitive to change [107], and additional outcomes, such as the proportion of children who were in the control or intervention group who became overweight at the end of the study period, which may have been more applicable to judge the downstream efficacy of the behavioural interventions, were not always available.

The observed small effect of MVPA in favour of the control group suggests that interventions promoting MVPA may not be effective, and this supports previous research that also highlighted the varying effectiveness of school-based interventions at improving MVPA [33,38]. Many of the interventions included in the current review were multi-component interventions and targeted a change in PA. Previous reviews have also attempted to establish what components in obesity interventions work [104,108,109]; however, only general findings were revealed, and the reviews were not solely focused on primary-school children. Within the literature we reviewed, there were no associations found between intervention components and effectiveness in the multi-component interventions, and it was not possible to determine which were the most effective components within these interventions. In line with the findings by a recent review [110], it is possible that effective multi-component interventions were influenced by a combination of intervention components. Thus, the effect of combining components on intervention effectiveness ought to be the topic of further research.

Multi-component interventions that incorporate diet, PA and SB-change components were not as effective as the single-component interventions at changing obesity-related behaviours in primary-school children. This is in contrast to the findings of a systematic review [108] that found that multi-component interventions (nutrition and PA), a single nutrition intervention and TV reduction were equally effective at achieving weight reduction in children and adolescents. However, in the Katz et al. [108] review, only a few studies (*n* = 8) were presented to adequately evaluate these strategies, and the robustness of these findings is limited due to the high degree of heterogeneity. More recently, a systematic review investigating interventions for the prevention of childhood obesity found low-certainty evidence that a combination of a diet and PA intervention reduced BMI z-score compared to the control group [45]. In contrast to the present review, Brown et al. [45] had a broader scope of included interventions, including those from the home and community, as well as the school setting. The efficiency of combined diet and PA school-based interventions to prevent obesity remains equivocal. For the current body of evidence, interventions based only on dietary improvement components were not associated with any increased efficacy in relation to nutrition behaviour, and this was consistent with other reviews [45,110]. The lack of effect observed for nutritional interventions may be construed by poor adherence to dietary interventions or the intricate interplay of intervention components.

No previous research has explored whether obesity-related outcomes were altered by theoretically driven school-based interventions solely in primary-school children. Over half of the included studies in this review did not specify the use of a behaviour change model or theory, and subgroup analyses inferred an effect on BMI z-score for interventions that were underpinned by behaviour change theory; however, although significant, the effect size was small. In most of the included studies that included a behaviour change model or theory, often a theoretical framework was only stated, rather than something that was used specifically in the intervention formulation which is highlighted in previous research [111]. Our review found no clear trend regarding a theory-based approach being more effective than interventions that were not based on a specific theory framework. In line with the current review, a previous review also found an insufficient amount of evidence that interventions underpinned by theory were more effective than those with no specified theory in after-school PA interventions [42].

The lack of a clear link between using a behaviour change model or theory and intervention effectiveness could be in part due to differences in the implementation of the underpinning theory within the interventions. Schools confront many challenges in converting evidence-based interventions into routine practice, for example: funding, school climate, resources, teacher self-belief, curriculum demands and implementation support [112]. Many theories, frameworks and models have been implemented in the trials included in this review. However, few interventions stated theoretical fidelity, which prevents direct inferences being made between intervention effectiveness and the underpinning theory [113]. For example, a recent systematic review reported that several barriers exist in the adoption, application and the integration of new PA interventions in schools [114]. In order to face this challenge, future interventions need to establish the exact links from theory to implementation as poor application of the theory may well be the reason of the lack of success in some interventions [115]. Understanding the implementation of interventions is essential, as interventions need to be planned, designed and delivered for use in real-world settings to have a population wide impact [116].

These findings reinforce that despite diversity of school-based interventions, the overall effect is promising in preventing weight gain among otherwise healthy children. Although difficult to compare outcomes between the studies included in this review due to the differing nature of the study design, target population and selected primary outcomes, the outcome of the analysis demonstrates the potential for school-based obesity prevention interventions. Nevertheless, it is challenging that so few studies were successful in increasing PA along with improving nutrition and/or reducing SB. Findings of the review should be interpreted in the context as the details of intervention characteristics varied considerably between interventions. Due to the considerable heterogeneity across paediatric obesity prevention interventions, with regards to certain interventions used (e.g., number, type and length), behavioural targets of the interventions and the measurement of outcomes, it is vital for authors to adopt an appropriate research design. Authors should provide adequate detail about their treatment strategies, theoretical basis and components and intensity of the interventions, as well as any implementation and assessment of programme fidelity, as this may be a promising approach for future intervention attempts.

Though school-based interventions have been proposed as being the most promising setting to tackle childhood obesity [31], the observed small effect exemplifies the difficulties and challenges positively impacting children’s obesity-related behaviours through the school setting. More research is required in the field on the impact of these interventions for long-term (e.g., more than one academic school year) obesity-related behaviour change. Few studies provided sufficient information for meta-analysis, and in some cases, it was necessary to rely on authors’ reporting of significant or non-significant effects on the interventions. Thus, these future studies should consider assessing a range of behaviours using validated objective measures and use standardised reporting of key outcomes (e.g., nutrition, sedentary and PA changes). Further research is required of school-based interventions in lower-income countries. From currently available evidence, it appears that long-term impact (e.g., more than one academic school year) of primary school-based interventions on maintenance of obesity-related behaviours needs further examination along with methodological rigor in the description and measurement of the target behaviours.

Collectively, the findings from this review provide a comprehensive evaluation of the literature on the impact of interventions on lifestyle behaviours deemed essential in the prevention of childhood obesity.

### Strengths and Limitations

This review contributes to the existing evidence base, as it is the first systematic review to assess the effectiveness of school-based interventions targeting obesity-related behaviours in primary-school children. The findings should be interpreted with caution considering the following limitations. First, the high level of heterogeneity detected across the included studies, which is a common finding amongst multi-component obesity interventions, limits the robustness of these findings. Pooled results indicate high levels of inconsistency across the included RCTs, and the majority of the analyses remained largely unexplained despite a large set of planned subgroup analyses. Therefore, the inconsistency is likely the result of variability in participants, settings, intervention components, outcomes and trial design. Heterogeneity of outcome measures is combined by heterogeneity of intervention methods, which consequently meant that extracting the data and findings was quite challenging.

Studies included in this review were restricted to English full-text publications, and it is therefore possible some relevant non-English studies were overlooked. Researchers had to rely on limited descriptions of interventions to classify the studies, and precisely assessing the contents of some interventions was difficult due to inconsistent reporting. The review did not investigate whether studies were powered using behavioural outcomes, and future systematic reviews should consider whether interventions were powered for the behavioural outcome. Although the overall systematic review identified 48 studies, when sub-group analyses were undertaken, included studies were limited (i.e., based on 1–15 studies), and as a result, it was not possible to examine whether the findings of potentially effective intervention components were influenced by device-based or self-reported measurements. Since the present review only included RCTs and cRCTs, these will, as a result, not address any complex interplay between behaviours and real-world settings. Nevertheless, the results of RCT study designs are valuable and are considered the gold standard [117]. Lastly, while the review attempted to separate out the most effective components, several studies comprised numerous complementary components that were not assessed individually.

This review is one of the first to use meta-analyses and subgroup analyses to systematically review several studies and analyse the potentially effective components of school-based interventions for preventing obesity in primary-school children.

## 5. Conclusions

Although a small significant intervention effect was found between groups in BMI z-scores, overall the findings were inconsistent, and the heterogeneity observed across all outcomes was not explained by subgrouping. Furthermore, the meta-analyses of the included interventions to prevent childhood obesity were inconclusive regarding MVPA, SB, nutrition behaviour and for BMI kg/m^2^ compared with the control condition. This review highlights the need to better understand how implementation of multi-component interventions deals with various intervention targets—perhaps it is just too much for schools to take on. It is important that future research investigates whether consecutive rather than concurrent sequencing of delivery is better. To conclude, the chance of success may be greater in a single-component intervention that only targets one obesity-related behaviour. It is important policy makers continue to recognise the school setting as a vehicle for tackling childhood obesity.

## Figures and Tables

**Table 2:** Effectiveness of interventions at changing obesity-related behaviours and BMI/BMI z-score.

**Outcome 1: MVPA (mins/day)**	**Studies (*n*=)**	**INT (*n*=)**	**C (*n*=)**	**I** **^2^**	**MD (95% CI)**
Device-measured	16	4523	3921	41%	1.53 (0.49, 2.57)
Self-reported	2	372	361	0%	12.37 (8.51, 16.22)
MVPA min/day (≤6 months)	4	280	285	0%	4.84 (0.81, 8.88)
MVPA min/day (>6 months)	14	4546	3997	74%	1.89 (0.09, 3.40)
Theory-based	9	3481	3102	74%	2.19 (0.04, 4.34)
No theory	9	1414	1266	49%	2.15 (−0.53, 4.82)
**Outcome 2: SB (mins/day)**	**Studies (*n*=)**	**INT (*n*=)**	**C (*n*=)**	**I** **^2^**	**MD (95% CI)**
Device-measured	11	3966	3456	55%	−0.91 (−2.30, 0.48)
Self-reported	1	40	52	N/A	1.40 (−25.95, 28.75)
ST min/day (≤6 months)	2	186	192	0%	1.29 (−5.67, 8.24)
ST min/day (>6 months)	10	3820	3316	59%	−1.00 (−2.42, 0.42)
Theory-based	6	3091	2691	20%	−0.46 (−1.93, 1.02)
No theory	5	915	817	65%	−4.51 (−8.68, −0.34)
**Outcome 3: BMI z-score**	**Studies (*n*=)**	**INT (*n*=)**	**C (*n*=)**	**I** **^2^**	**MD (95% CI)**
Only targeting one outcome	5	1465	1565	68%	−0.05 (−0.10, 0.01)
Targeting > 1 outcome	11	5935	5386	57%	−0.04 (−0.08, 0.00)
≤6 months	2	293	314	52%	−0.02 (−0.15, 0.10)
>6 months	14	7107	6637	61%	−0.04 (−0.08, −0.01)
Theory-based	6	3253	3287	68%	−0.08 (−0.13, −0.02)
No theory	10	4147	3664	47%	−0.02 (−0.06, 0.02)
**Outcome 4: BMI (kg/m** **^2^)**	**Studies (*n*=)**	**INT (*n*=)**	**C (*n*=)**	**I** **^2^**	**MD (95% CI)**
Only targeting one outcome	8	2451	2469	12%	−0.04 (−0.23, 0.16)
Targeting > 1 outcome	12	4700	4174	67%	−0.04 (−0.14, 0.06)
≤6 months	5	491	502	42%	−0.07 (−0.35, 0.22)
>6 months	15	6660	6141	59%	−0.03 (−0.13, 0.06)
Theory-based	6	2596	2596	75%	−0.10 (−0.22, 0.02)
No theory	14	4555	4047	30%	0.04 (−0.09, 0.18)

Abbreviations: INT = intervention, C = control, MVPA = moderate-to-vigorous physical activity, SB = sedentary behaviour, ST = sedentary time, MD = mean difference, BMI = body mass index.

## Data Availability

The dataset(s) supporting the conclusions of this article are included within the article (and Supplementary Materials).

## Supplementary Materials

The following are available online at https://www.mdpi.com/article/10.3390/children8060489/s1, Table S1: PRISMA checklist. Table S2, Search Strategy. Table S3 Characteristics of included studies.

## References

[1] Bhadoria A.S., Sahoo K., Sahoo B., Choudhury A.K., Sofi N.Y., Kumar C.A. (2015). Childhood obesity: Causes and consequences. J. Fam. Med. Prim. Care.

[2] NCD Risk Factor Collaboration (NCD-RisC) (2017). Worldwide trends in body-mass index, underweight, overweight, and obesity from 1975 to 2016: A pooled analysis of 2416 population-based measurement studies in 128 9 million children, adolescents, and adults. Lancet.

[3] Tremmel M., Gerdtham U.-G., Nilsson P.M., Saha S. (2017). Economic Burden of Obesity: A Systematic Literature Review. Int. J. Environ. Res. Public Health.

[4] Hill J.O., Wyatt H.R., Peters J.C. (2012). Energy Balance and Obesity. Circulation.

[5] Romieu I., Dossus L., Barquera S., Blottière H.M., Franks P.W., Gunter M., Hwalla N., Hursting S.D., Leitzmann M., Margetts B. (2017). Energy balance and obesity: What are the main drivers?. Cancer Causes Control.

[6] Thivel D., Aucouturier J., Doucet É., Saunders T., Chaput J.-P. (2013). Daily energy balance in children and adolescents. Does energy expenditure predict subsequent energy intake?. Appetite.

[7] Ekelund U. (2012). Moderate to Vigorous Physical Activity and Sedentary Time and Cardiometabolic Risk Factors in Children and Adolescents. JAMA.

[8] Andersen Andersen L.B., Harro M., Sardinha L.B., Froberg K., Ekelund U., Brage S., Anderssen S.A. (2006). Physical activity and clustered cardiovascular risk in children: A cross-sectional study (The European Youth Heart Study). Lancet.

[9] Hills A.P., King N.A., Armstrong T.P. (2007). The Contribution of Physical Activity and Sedentary Behaviours to the Growth and Development of Children and Adolescents. Sports Med..

[10] Janssen I., LeBlanc A.G. (2010). Systematic review of the health benefits of physical activity and fitness in school-aged children and youth. Int. J. Behav. Nutr. Phys. Act..

[11] Kim J., Lim H. (2019). Nutritional Management in Childhood Obesity. J. Obes. Metab. Syndr..

[12] Luque V., Escribano J., Closa-Monasterolo R., Zaragoza-Jordana M., Ferré N., Grote V., Koletzko B., Totzauer M., Verduci E., ReDionigi A. (2018). Unhealthy Dietary Patterns Established in Infancy Track to Mid-Childhood: The EU Childhood Obesity Project. J. Nutr..

[13] Ng M., Fleming T., Robinson M., Thomson B., Graetz N., Margono C., Mullany E.C., Biryukov S., Abbafati C., Abera S.F. (2014). Global, regional, and national prevalence of overweight and obesity in children and adults during 1980–2013: A systematic analysis for the Global Burden of Disease Study 2013. Lancet.

[14] Ambrosini G. (2014). Childhood dietary patterns and later obesity: A review of the evidence. Proc. Nutr. Soc..

[15] Gibbs B.B., Hergenroeder A.L., Katzmarzyk P., Lee I.-M., Jakicic J.M. (2015). Definition, Measurement, and Health Risks Associated with Sedentary Behavior. Med. Sci. Sports Exerc..

[16] Young D.R., Hivert M.-F., Alhassan S., Camhi S.M., Ferguson J.F., Katzmarzyk P., Lewis C.E., Owen N., Perry C.K., Siddique J. (2016). Sedentary Behavior and Cardiovascular Morbidity and Mortality: A Science Advisory from the American Heart Association. Circulation.

[17] Mann K., Howe L.D., Basterfield L., Parkinson K.N., Pearce M.S., Reilly J.K., Adamson A.J., Janssen X. (2017). Longitudinal study of the associations between change in sedentary behavior and change in adiposity during childhood and adolescence: Gateshead Millennium Study. Int. J. Obes..

[18] Aubert S., Barnes J.D., Abdeta C., Abi Nader P., Adeniyi A.F., Aguilar-Farias N., Tenesaca D.S.A., Bhawra J., Brazo-Sayavera J., Cardon G. (2018). Global Matrix 3.0 Physical Activity Report Card Grades for Children and Youth: Results and Analysis From 49 Countries. J. Phys. Act. Health.

[19] Timperio A., Salmon J., Ball K., Baur L.A., Telford A., Jackson M., Salmon L., Crawford D. (2008). Family physical activity and sedentary environments and weight change in children. Pediatr. Obes..

[20] Dooyema C.A., Belay B., Blanck H.M. (2017). Implementation of Multisetting Interventions to Address Childhood Obesity in Diverse, Lower-Income Communities: CDC’s Childhood Obesity Research Demonstration Projects. Prev. Chronic Dis..

[21] Hancox R.J., Milne B.J., Poulton R. (2005). Association of television viewing during childhood with poor educational achievement. Arch. Pediatr. Adolesc. Med..

[22] Prentice-Dunn H., Prentice-Dunn S. (2012). Physical activity, sedentary behavior, and childhood obesity: A review of cross-sectional studies. Psychol. Health Med..

[23] Craigie A.M., Lake A.A., Kelly S., Adamson A.J., Mathers J.C. (2011). Tracking of obesity-related behaviours from childhood to adulthood: A systematic review. Maturitas.

[24] Llewellyn A., Simmonds M.C., Owen C.G., Woolacott N. (2016). Childhood obesity as a predictor of morbidity in adulthood: A systematic review and meta-analysis. Obes. Rev..

[25] Mikkilä V., Räsänen L., Raitakari O.T., Marniemi J., Pietinen P., Rönnemaa T., Viikari J. (2007). Major dietary patterns and cardiovascular risk factors from childhood to adulthood. The Cardiovascular Risk in Young Finns Study. Br. J. Nutr..

[26] Singh A.S., Mulder C., Twisk J.W., Van Mechelen W., Chinapaw M.J. (2008). Tracking of childhood overweight into adulthood: A systematic review of the literature. Obes. Rev..

[27] Jones R.A., Hinkley T., Okely A., Salmon J. (2013). Tracking Physical Activity and Sedentary Behavior in Childhood. Am. J. Prev. Med..

[28] Flynn M.A.T., McNeil D.A., Maloff B., Mutasingwa D., Wu M., Ford C., Tough S.C. (2006). Reducing obesity and related chronic disease risk in children and youth: A synthesis of evidence with ’best practice’ recommendations. Obes. Rev..

[29] Kelishadi R., Azizi-Soleiman F. (2014). Controlling childhood obesity: A systematic review on strategies and challenges. J. Res. Med. Sci..

[30] Fox K.R. (2004). Childhood obesity and the role of physical activity. J. R. Soc. Promot. Health.

[31] Kriemler S., Meyer U., Martin E., van Sluijs E., Andersen L.B., Martin B.W. (2011). Effect of school-based interventions on physical activity and fitness in children and adolescents: A review of reviews and systematic update. Br. J. Sports Med..

[32] Hillier-Brown F.C., Bambra C.L., Cairns J.-M., Kasim A., Moore H.J., Summerbell C.D. (2014). A systematic review of the effectiveness of individual, community and societal level interventions at reducing socioeconomic inequalities in obesity amongst children. BMC Public Health.

[33] Dobbins M., De Corby K., Robeson P., Husson H., Tirilis D. (2009). School-based physical activity programs for promoting physical activity and fitness in children and adolescents aged 6-18. Cochrane Database Syst. Rev..

[34] Gomes T.N., Katzmarzyk P.T., Hedeker D., Fogelholm M., Standage M., Onywera V., Lambert E., Tremblay M.S., Chaput J.-P., Tudor-Locke C. (2017). Correlates of compliance with recommended levels of physical activity in children. Sci. Rep..

[35] Cottona W., Dudleyb D., Peraltaa L., Werkhovena T. (2020). The effect of teacher-delivered nutrition education programs on elementary-aged students: An updated systematic review and meta-analysis. Prev. Med. Rep..

[36] Murimi M.W., Moyeda-Carabaza A.F., Nguyen B., Saha S., Amin R., Njike V. (2018). Factors that contribute to effective nutrition education interventions in children: A systematic review. Nutr. Rev..

[37] National Institute for Public Health, (NICE) Promoting Physical Activity, Active Play and Sport for Pre-School and School-Age Children and Young People in Family, Pre-School, School and Community Settings. NICE 2009. https://www.nice.org.uk/PH17.

[38] Metcalf B., Henley W., Wilkin T. (2012). Effectiveness of intervention on physical activity of children: Systematic review and meta-analysis of controlled trials with objectively measured outcomes (EarlyBird 54). BMJ.

[39] Parrish A.-M., Okely A., Stanley R., Ridgers N.D. (2013). The Effect of School Recess Interventions on Physical Activity. Sports Med..

[40] Lonsdale C., Rosenkranz R.R., Peralta L.R., Bennie A., Fahey P., Lubans D.R. (2013). A systematic review and meta-analysis of interventions designed to increase moderate-to-vigorous physical activity in school physical education lessons. Prev. Med..

[41] Demetriou Y., Gillison F., McKenzie T.L. (2017). After-school physical activity interventions on child and adolescent physical activity and health: A review of reviews. Adv. Phys. Educ..

[42] Mears R., Jago R. (2016). Effectiveness of after-school interventions at increasing moderate-to-vigorous physical activity levels in 5- to 18-year olds: A systematic review and meta-analysis. Br. J. Sports Med..

[43] Van Grieken A., Ezendam N.P.M., Paulis W.D., Van Der Wouden J.C., Raat H. (2012). Primary prevention of overweight in children and adolescents: A meta-analysis of the effectiveness of interventions aiming to decrease sedentary behaviour. Int. J. Behav. Nutr. Phys. Act..

[44] Muthuri S.K., Wachira L.-J.M., Leblanc A.G., Francis C.E., Sampson M., Onywera V.O., Tremblay M.S. (2014). Temporal Trends and Correlates of Physical Activity, Sedentary Behaviour, and Physical Fitness among School-Aged Children in Sub-Saharan Africa: A Systematic Review. Int. J. Environ. Res. Public Health.

[45] Brown T., Moore T.H., Hooper L., Gao Y., Zayegh A., Ijaz S., Elwenspoek M., Foxen S.C., Magee L., O’Malley C. (2019). Interventions for preventing obesity in children. Cochrane Database Syst. Rev..

[46] Brown T., Summerbell C. (2009). Systematic review of school-based interventions that focus on changing dietary intake and physical activity levels to prevent childhood obesity: An update to the obesity guidance produced by the National Institute for Health and Clinical Excellence. Obes. Rev..

[47] Kelishadi R., Roufarshbaf M., Soheili S., Payghambarzadeh F., Masjedi M. (2017). Association of Childhood Obesity and the Immune System: A Systematic Review of Reviews. Child. Obes..

[48] Moher D., Liberati A., Tetzlaff J., Altman D.G., The PRISMA Group (2009). Preferred reporting items for systematic reviews and meta-analyses: The PRISMA statement. PLoS Med..

[49] Yildirim M., van Stralen M., Chinapaw M.J.M., Brug J., Van Mechelen W., Twisk J.W.R., Velde S.J.T. (2011). For whom and under what circumstances do school-based energy balance behavior interventions work? Systematic review on moderators. Pediatr. Obes..

[50] Hoffmann T.C., Glasziou P.P., Boutron I., Milne R., Perera R., Moher D., Michie S. (2014). Better reporting of interventions: Template for intervention description and replication (TIDieR) checklist and guide. BMJ.

[51] Hoffmann T.C., Oxman A.D., Ioannidis J.P., Moher D., Lasserson T.J., I Tovey D., Stein K., Sutcliffe K., Ravaud P., Altman D.G. (2017). Enhancing the usability of systematic reviews by improving the consideration and description of interventions. BMJ.

[52] Higgins J.P.T., Altman D.G., Gøtzsche P.C., Jüni P., Moher D., Oxman A.D., Savović J., Schulz K.F., Weeks L., Sterne J.A.C. (2011). The Cochrane Collaboration’s tool for assessing risk of bias in randomised trials. BMJ.

[53] Higgins J.P.T., Thompson S.G. (2002). Quantifying heterogeneity in a meta-analysis. Stat. Med..

[54] Ahmed I., Sutton A.J., Riley R.D. (2012). Assessment of publication bias, selection bias, and unavailable data in meta-analyses using individual participant data: A database survey. BMJ.

[55] Sterne J., Becker B.J., Egger M. (2006). The Funnel Plot. Publ. Bias Meta-Anal..

[56] Lynch B.A., Gentile N., Maxson J., Quigg S., Swenson L., Kaufman T. (2016). Elementary School–Based Obesity Intervention Using an Educational Curriculum. J. Prim. Care Community Health.

[57] Marcus C., Nyberg G., Nordenfelt A., Karpmyr M., Kowalski J., Ekelund U. (2009). A 4-year, cluster-randomized, controlled childhood obesity prevention study: STOPP. Int. J. Obes..

[58] Llargues E., Franco R., Recasens A., Nadal A., Vila M., Pérez M.J., Manresa J.M., Recasens I., Salvador G., Serra J. (2011). Assessment of a school-based intervention in eating habits and physical activity in school children: The AVall study. J. Epidemiol. Community Health.

[59] Howe C.A., Harris R.A., Gutin B. (2011). A 10-Month Physical Activity Intervention Improves Body Composition in Young Black Boys. J. Obes..

[60] Amini M., Djazayery A., Majdzadeh R., Taghdisi M.-H., Sadrzadeh-Yeganeh H., Abdollahi Z., Hosseinpour-Niazi N., Chamari M., Nourmohammadi M. (2016). A School-Based Intervention to Reduce Excess Weight in Overweight and Obese Primary School Students. Biol. Res. Nurs..

[61] Drummy C., Murtagh E., McKee D.P., Breslin G., Davison G.W., Murphy M.H. (2016). The effect of a classroom activity break on physical activity levels and adiposity in primary school children. J. Paediatr. Child Health.

[62] Telford R.M., Olive L.S., Cochrane T., Davey R., Telford R. (2016). Outcomes of a four-year specialist-taught physical education program on physical activity: A cluster randomized controlled trial, the LOOK study. Int. J. Behav. Nutr. Phys. Act..

[63] Kobel S., Wirt T., Schreiber A., Kesztyüs D., Kettner S., Erkelenz N., Wartha O., Steinacker J.M. (2014). Intervention Effects of a School-Based Health Promotion Programme on Obesity Related Behavioural Outcomes. J. Obes..

[64] Kobel S., Lämmle C., Wartha O., Kesztyüs D., Wirt T., Steinacker J.M. (2016). Effects of a Randomised Controlled School-Based Health Promotion Intervention on Obesity Related Behavioural Outcomes of Children with Migration Background. J. Immigr. Minor. Health.

[65] Adab P., Pallan M.J., Lancashire E.R., Hemming K., Frew E., Barrett T., Bhopal R., E Cade J., Canaway A., Clarke J. (2018). Faculty Opinions recommendation of Effectiveness of a childhood obesity prevention programme delivered through schools, targeting 6 and 7 year olds: Cluster randomised controlled trial (WAVES study). BMJ.

[66] Anderson E.L., Howe L.D., Kipping R.R., Campbell R., Jago R., Noble S.M., Wells S., Chittleborough C., Peters T., A Lawlor D. (2016). Long-term effects of the Active for Life Year 5 (AFLY5) school-based cluster-randomised controlled trial. BMJ Open.

[67] Engelen L., Bundy A.C., Naughton G., Simpson J.M., Bauman A., Ragen J., van der Ploeg H.P. (2013). Increasing physical activity in young primary school children—it’s child’s play: A cluster randomised controlled trial. Prev. Med..

[68] Fairclough S.J., Hackett A.F., Davies I.G., Gobbi R., A Mackintosh K., Warburton G.L., Stratton G., Van Sluijs E.M., Boddy L.M. (2013). Promoting healthy weight in primary school children through physical activity and nutrition education: A pragmatic evaluation of the CHANGE! randomised intervention study. BMC Public Health.

[69] Li B., Pallan M., Liu W.J., Hemming K., Frew E., Lin R., Martin J., Zanganeh M., Hurley K., Cheng K.K. (2019). The CHIRPY DRAGON intervention in preventing obesity in Chinese primary-school--aged children: A cluster-randomised controlled trial. PLoS Med..

[70] Madsen K., Linchey J., Gerstein D., Ross M., Myers E., Brown K., Crawford P. (2015). Energy balance 4 kids with play: Results from a two-year cluster-randomized trial. Child. Obes..

[71] Martínez-Vizcaíno V., Sánchez-López M., Notario-Pacheco B., Salcedo-Aguilar F., Solera-Martínez M., Franquelo-Morales P., López-Martínez S., García-Prieto J.C., Arias-Palencia N., Torrijos-Niño C. (2014). Gender differences on effectiveness of a school-based physical activity intervention for reducing cardiometabolic risk: A cluster randomized trial. Int. J. Behav. Nutr. Phys. Act..

[72] Nathan N.K., Sutherland R.L., Hope K., McCarthy N.J., Pettett M., Elton B., Jackson R., Trost S.G., Lecathelinais C., Reilly K. (2020). Implementation of a School Physical Activity Policy Improves Student Physical Activity Levels: Outcomes of a Cluster-Randomized Controlled Trial. J. Phys. Act. Health.

[73] Resaland G.K., Aadland E., Moe V.F., Aadland K.N., Skrede T., Stavnsbo M., Suominen L., Steene-Johannessen J., Glosvik Ø., Andersen J.R. (2016). Effects of physical activity on schoolchildren’s academic performance: The Active Smarter Kids (ASK) cluster-randomized controlled trial. Prev. Med..

[74] Seljebotn P.H., Skage I., Riskedal A., Olsen M., Kvalø S.E., Dyrstad S.M. (2019). Physically active academic lessons and effect on physical activity and aerobic fitness. The Active School study: A cluster randomized controlled trial. Prev. Med. Rep..

[75] Sacchetti R., Ceciliani A., Garulli A., Dallolio L., Beltrami P., Leoni E. (2013). Effects of a 2-Year School-Based Intervention of Enhanced Physical Education in the Primary School. J. Sch. Health.

[76] Tarro L., Llauradó E., Albaladejo R., Moriña D., Arija V., Solà R., Giralt M. (2014). A primary-school-based study to reduce the prevalence of childhood obesity—The EdAl (Educació en Alimentació) study: A randomized controlled trial. Trials.

[77] Habib-Mourad C., Ghandour L.A., Maliha C., Awada N., Dagher M., Hwalla N. (2020). Impact of a one-year school-based teacher-implemented nutrition and physical activity intervention: Main findings and future recommendations. BMC Public Health.

[78] Santina T., Beaulieu D., Gagné C., Guillaumie L. (2021). Tackling childhood obesity through a school-based physical activity programme: A cluster randomised trial. Int. J. Sport Exerc. Psychol..

[79] Williamson D.A., Champagne C.M., Harsha D.W., Han H., Martin C.K., Newton R., Sothern M.S., Stewart T.M., Webber L.S., Ryan D.H. (2012). Effect of an Environmental School-Based Obesity Prevention Program on Changes in Body Fat and Body Weight: A Randomized Trial. Obesity.

[80] Bacardi-Gascon M., Pérez-Morales M., Jiménez-Cruz A. (2012). A six month randomized school intervention and an 18-month follow-up intervention to prevent childhood obesity in Mexican elementary schools. Nutr. Hosp..

[81] Efstathiou N.T., Risvas G.S., Theodoraki E.-M.M., Galanaki E.P., Zampelas A.D. (2016). Health education: Effects on classroom climate and physical activity. Health Educ. J..

[82] Wells N.M., Myers B.M., Henderson C.R. (2014). School gardens and physical activity: A randomized controlled trial of low-income elementary schools. Prev. Med..

[83] Donnelly J.E., Greene J.L., Gibson C.A., Smith B.K., Washburn R.A., Sullivan D.K., DuBose K., Mayo M.S., Schmelzle K.H., Ryan J.J. (2009). Physical Activity Across the Curriculum (PAAC): A randomized controlled trial to promote physical activity and diminish overweight and obesity in elementary school children. Prev. Med..

[84] Farmer V.L., Williams S.M., I Mann J., Schofield G., McPhee J.C., Taylor R.W. (2017). The effect of increasing risk and challenge in the school playground on physical activity and weight in children: A cluster randomised controlled trial (PLAY). Int. J. Obes..

[85] Lau P.W.C., Wang J.J., Maddison R. (2016). A Randomized-Controlled Trial of School-Based Active Videogame Intervention on Chinese Children’s Aerobic Fitness, Physical Activity Level, and Psychological Correlates. Games Health J..

[86] O’Leary M., Rush E., Lacey S., Burns C., Coppinger T. (2018). Project Spraoi: Two year outcomes of a whole school physical activity and nutrition intervention using the RE-AIM framework. Ir. Educ. Stud..

[87] Angelopoulos P.D., Milionis H.J., Grammatikaki E., Moschonis G., Manios Y. (2009). Changes in BMI and blood pressure after a school based intervention: The CHILDREN study. Eur. J. Public Health.

[88] Rosário R., Araújo A., Padrão P., Lopes O., Moreira A., Pereira B., Moreira P. (2016). Health Promotion Intervention to Improve Diet Quality in Children. Health Promot. Prcat..

[89] Siegrist M., Lammel C., Haller B., Christle J., Halle M. (2011). Effects of a physical education program on physical activity, fitness, and health in children: The JuvenTUM project. Scand. J. Med. Sci. Sports.

[90] Viggiano E., Viggiano A., Di Costanzo A., Viggiano A., Viggiano A., Andreozzi E., Romano V., Vicidomini C., Di Tuoro D., Gargano G. (2018). Healthy lifestyle promotion in primary schools through the board game Kaledo: A pilot cluster randomized trial. Eur. J. Nucl. Med. Mol. Imaging.

[91] Kocken P.L., Scholten A.-M., Westhoff E., De Kok B.P.H., Taal E.M., Goldbohm R.A. (2016). Effects of a Theory-Based Education Program to Prevent Overweightness in Primary School Children. Nutrients.

[92] Kain J., Concha F., Moreno L., Leyton B. (2014). School-Based Obesity Prevention Intervention in Chilean Children: Effective in Controlling, but not Reducing Obesity. J. Obes..

[93] Scherr R.E., Linnell J.D., Dharmar M., Beccarelli L.M., Bergman J.J., Briggs M., Brian K.M., Feenstra G., Hillhouse J.C., Keen C.L. (2017). A Multicomponent, School-Based Intervention, the Shaping Healthy Choices Program, Improves Nutrition-Related Outcomes. J. Nutr. Educ. Behav..

[94] Rausch Herscovici C., Kovalskys I., De Gregorio M.J. (2013). Gender differences and a school-based obesity prevention program in Argentina: A randomized trial. Rev. Panam. Salud Publica.

[95] Xu F., Ware R.S., Leslie E., Tse L.A., Wang Z., Li J., Wang Y. (2015). Effectiveness of a randomized controlled lifestyle intervention to prevent obesity among Chinese primary school students: CLICK-obesity study. PLoS ONE.

[96] Nickel N.C., Doupe M., Enns J.E., Brownell M., Sarkar J., Chateau D., Burland E., Chartier M., Katz A., Crockett L. (2021). Differential effects of a school-based obesity prevention program: A cluster randomized trial. Matern. Child Nutr..

[97] Ford P.A., Perkins G., Swaine I. (2012). Effects of a 15-week accumulated brisk walking programme on the body composition of primary school children. J. Sports Sci..

[98] Khan N.A., Raine L.B., Drollette E.S., Scudder M.R., Pontifex M.B., Castelli D.M., Donovan S., Evans E.M., Hillman C.H. (2014). Impact of the FITKids Physical Activity Intervention on Adiposity in Prepubertal Children. Pediatrics.

[99] Brandstetter S., Klenk J., Berg S., Galm C., Fritz M., Peter R., Prokopchuk D., Steiner R.P., Wartha O., Steinacker J.M. (2012). Overweight Prevention Implemented by Primary School Teachers: A Randomised Controlled Trial. Obes. Facts.

[100] Lloyd J., Creanor S., Logan S., Green C., Dean S., Hillsdon M., Abraham C., Tomlinson R., Pearson V., Taylor R.S. (2018). Effectiveness of the Healthy Lifestyles Programme (HeLP) to prevent obesity in UK primary-school children: A cluster randomised controlled trial. Lancet Child Adolesc. Health.

[101] Liu Z., Li Q., Maddison R., Ni Mhurchu C., Jiang Y., Wei D.-M., Cheng L., Cheng Y., Wang D., Wang H.-J. (2019). A School-Based Comprehensive Intervention for Childhood Obesity in China: A Cluster Randomized Controlled Trial. Child. Obes..

[102] Bere E., Klepp K.-I., Øverby N.C. (2014). Free school fruit: Can an extra piece of fruit every school day contribute to the prevention of future weight gain? A cluster randomized trial. Food Nutr. Res..

[103] Llauradó E., Tarro L., Moriña D., Queral R., Giralt M., Solà R. (2014). EdAl-2 (Educació en Alimentació) programme: Reproducibility of a cluster randomised, interventional, primary-school-based study to induce healthier lifestyle activities in children. BMJ Open.

[104] Luttikhuis H.O., Baur L., Jansen H., Shrewsbury V.A., O’Malley C., Stolk R., Summerbell C. (2009). Interventions for treating obesity in children. Cochrane Database Syst. Rev..

[105] Mead E., Brown T., Rees K., Azevedo L., Whittaker V., Jones D., Olajide J., Mainardi G.M., Corpeleijn E., O’Malley C. (2017). Diet, physical activity and behavioural interventions for the treatment of overweight or obese children from the age of 6 to 11 years. Cochrane Database Syst. Rev..

[106] Zolotarjova J., Velde G.T., Vreugdenhil A.C.E. (2018). Effects of multidisciplinary interventions on weight loss and health outcomes in children and adolescents with morbid obesity. Obes. Rev..

[107] Kolotourou M., Radley D., Chadwick P., Smith L., Orfanos S., Kapetanakis V., Singhal A., Cole T., Sacher P.M. (2013). Is BMI Alone a Sufficient Outcome to Evaluate Interventions for Child Obesity?. Child. Obes..

[108] Katz D.L., O’Connell M., Njike V.Y., Yeh M.-C., Nawaz H. (2008). Strategies for the prevention and control of obesity in the school setting: Systematic review and meta-analysis. Int. J. Obes..

[109] Feng L., Wei D.M., Lin S.T., Maddison R., Ni Mhurchu C., Jiang Y., Wang H.J. (2017). Systematic review and meta-analysis of school-based obesity interventions in mainland China. PLoS ONE.

[110] Liu Z., Xu H.-M., Wen L.-M., Peng Y.-Z., Lin L.-Z., Zhou S., Li W.-H., Wang H.-J. (2019). A systematic review and meta-analysis of the overall effects of school-based obesity prevention interventions and effect differences by intervention components. Int. J. Behav. Nutr. Phys. Act..

[111] Panter J., Andersen P.T., Aro A.R., Samara A. (2018). Obesity Prevention: A Systematic Review of Setting-Based Interventions from Nordic Countries and the Netherlands. J. Obes..

[112] Kearns N.E., Kleinert J.O., Dupont-Versteegden E.E. (2019). Implementing Multilevel School-Based Physical Activity Interventions Using Core Implementation Components Model. J. Sch. Health.

[113] Pérez D., Van Der Stuyft P., Zabala M.D.C., Castro M., Lefèvre P. (2015). A modified theoretical framework to assess implementation fidelity of adaptive public health interventions. Implement. Sci..

[114] Cassar S., Salmon J., Timperio A., Naylor P.-J., Van Nassau F., Ayala A.M.C., Koorts H. (2019). Adoption, implementation and sustainability of school-based physical activity and sedentary behaviour interventions in real-world settings: A systematic review. Int. J. Behav. Nutr. Phys. Act..

[115] Naylor P.-J., Nettlefold L., Race D., Hoy C., Ashe M.C., Higgins J.W., McKay H.A. (2015). Implementation of school based physical activity interventions: A systematic review. Prev. Med..

[116] Hailemariam M., Bustos T., Montgomery B., Barajas R., Evans L.B., Drahota A. (2019). Evidence-based intervention sustainability strategies: A systematic review. Implement. Sci..

[117] Victora C.G., Habicht J.P., Bryce J. (2004). Evidence-based public health: Moving beyond randomized trials. Am. J. Public Health.

