# Correction: Nally et al. The Effectiveness of School-Based Interventions on Obesity-Related Behaviours in Primary School Children: A Systematic Review and Meta-Analysis of Randomised Controlled Trials. *Children* 2021, *8*, 489

**DOI:** 10.3390/children11091092

**Published:** 2024-09-06

**Authors:** Sarah Nally, Angela Carlin, Nicole E. Blackburn, Judith S. Baird, Jo Salmon, Marie H. Murphy, Alison M. Gallagher

**Affiliations:** 1 Centre for Exercise Medicine, Physical Activity and Health, Sports and Exercise Sciences Research Institute, Jordanstown Campus, University of Ulster, Newtownabbey BT37 0QB, UK; nally-s@ulster.ac.uk (S.N.); a.carlin1@ulster.ac.uk (A.C.); mh.murphy@ulster.ac.uk (M.H.M.); 2 Centre for Health and Rehabilitation Technologies, Institute of Nursing and Health Research, School of Health Sciences, University of Ulster, Newtownabbey BT37 0QB, UK; ne.blackburn@ulster.ac.uk; 3 Nutrition Innovation Centre for Food and Health (NICHE), Biomedical Sciences Research Institute, Coleraine Campus, University of Ulster, Coleraine BT52 1SA, UK; baird-j7@ulster.ac.uk; 4 Institute for Physical Activity and Nutrition (IPAN), School of Exercise and Nutrition Sciences, Deakin University, Geelong 3217, Australia; jo.salmon@deakin.edu.au

We acknowledge an error in the BMI data in our publication [1] pointed out by Ginell et al. [2]. To address this, the authors undertook a comprehensive review of the original results. When corrected and reanalysed, no significant intervention effect was observed in children for BMI (kg/m^2^) compared with control and this is reflected in the amended manuscript submitted to the journal in July 2023. A detailed account of all the amendments to the original paper is included below:

The fifth-last and second-last sentences of the abstract should read as follows:

Thirty-seven studies were eligible for inclusion in a meta-analysis. The findings demonstrate that interventions in children when compared to controls resulted in a small positive treatment effect in MVPA in the control group (2.14; 95% CI = 0.77, 3.50). There was no significant effect on sedentary behaviour, energy intake, and fruit and vegetable intake, and BMI kg/m^2^. A small significant reduction was found between groups in BMI z-score (−0.04; 95% CI = −0.07, −0.01) in favour of the intervention.

The methodology regarding the meta-analysis (Section 2.6) has been amended to clarify that mean differences are reported (not standardised mean differences). The titles of Figures 3 and 4 have also been amended to reflect this.

Section 2.6 now reads as follows:

The effect sizes were determined (mean differences) reporting 95% CI for the difference between arms (intervention vs. control) for each of the three behavioural outcomes and for BMI, as implemented into Review Manager (RevMan) (*Version 5.3. Copenhagen: The Nordic Cochrane Centre, The Cochrane Collaboration, 2014*). Data were pooled to compare the post intervention mean differences between intervention vs control for each of the outcomes.

The updated captions for Figure 3 and Figure 4 now read as follows:

**Figure 3.** Difference between intervention and control groups of school-based interventions in primary school children at follow-up in BMI (kg/m^2^).

**Figure 4.** Difference between intervention and control groups of school-based interventions in primary school children in BMI z-score.

The number of studies included in Sections 3.4–3.6 has been updated from 38 to 37.

Following a full reanalysis, updated findings for outcome 3 (BMI z-score) and outcome 4 (BMI kg/m^2^) are presented in Section 3.8 and Table 2. The overall conclusions and take-home messages of the paper have not changed as a result of this reanalysis.

The updated Table 2 now reads as follows:

**Table 2:** Effectiveness of interventions at changing obesity-related behaviours and BMI/BMI z-score.

**Outcome 1: MVPA (mins/day)**	**Studies (*n*=)**	**INT (*n*=)**	**C (*n*=)**	**I** **^2^**	**MD (95% CI)**
Device-measured	16	4523	3921	41%	1.53 (0.49, 2.57)
Self-reported	2	372	361	0%	12.37 (8.51, 16.22)
MVPA min/day (≤6 months)	4	280	285	0%	4.84 (0.81, 8.88)
MVPA min/day (>6 months)	14	4546	3997	74%	1.89 (0.09, 3.40)
Theory-based	9	3481	3102	74%	2.19 (0.04, 4.34)
No theory	9	1414	1266	49%	2.15 (−0.53, 4.82)
**Outcome 2: SB (mins/day)**	**Studies (*n*=)**	**INT (*n*=)**	**C (*n*=)**	**I** **^2^**	**MD (95% CI)**
Device-measured	11	3966	3456	55%	−0.91 (−2.30, 0.48)
Self-reported	1	40	52	N/A	1.40 (−25.95, 28.75)
ST min/day (≤6 months)	2	186	192	0%	1.29 (−5.67, 8.24)
ST min/day (>6 months)	10	3820	3316	59%	−1.00 (−2.42, 0.42)
Theory-based	6	3091	2691	20%	−0.46 (−1.93, 1.02)
No theory	5	915	817	65%	−4.51 (−8.68, −0.34)
**Outcome 3: BMI z-score**	**Studies (*n*=)**	**INT (*n*=)**	**C (*n*=)**	**I** **^2^**	**MD (95% CI)**
Only targeting one outcome	5	1465	1565	68%	−0.05 (−0.10, 0.01)
Targeting > 1 outcome	11	5935	5386	57%	−0.04 (−0.08, 0.00)
≤6 months	2	293	314	52%	−0.02 (−0.15, 0.10)
>6 months	14	7107	6637	61%	−0.04 (−0.08, −0.01)
Theory-based	6	3253	3287	68%	−0.08 (−0.13, −0.02)
No theory	10	4147	3664	47%	−0.02 (−0.06, 0.02)
**Outcome 4: BMI (kg/m** **^2^)**	**Studies (*n*=)**	**INT (*n*=)**	**C (*n*=)**	**I** **^2^**	**MD (95% CI)**
Only targeting one outcome	8	2451	2469	12%	−0.04 (−0.23, 0.16)
Targeting > 1 outcome	12	4700	4174	67%	−0.04 (−0.14, 0.06)
≤6 months	5	491	502	42%	−0.07 (−0.35, 0.22)
>6 months	15	6660	6141	59%	−0.03 (−0.13, 0.06)
Theory-based	6	2596	2596	75%	−0.10 (−0.22, 0.02)
No theory	14	4555	4047	30%	0.04 (−0.09, 0.18)

Abbreviations: INT = intervention, C = control, MVPA = moderate-to-vigorous physical activity, SB = sedentary behaviour, ST = sedentary time, MD = mean difference, BMI = body mass index.

The updated Section 3.8 now reads as follows:

The overall effect size in 20 studies for BMI kg/m^2^ is summarised in Figure 3, and the overall effect size in 16 studies for BMI z-score is summarised in Figure 4. The quantitative synthesis of the interventions showed a non-significant reduction in BMI (−0.04 kg/m^2^; 95% CI =−0.13, 0.05) and a small significant reduction in BMI z-score (−0.04; 95% CI = −0.07, −0.01) compared with the control group. Sensitivity analysis was conducted in studies measuring change from baseline rather than follow-up data in BMI (kg/m^2^), removal of Ford et al. [97] = (−0.04; 95% CI = −0.13, 0.05). Subgroup analyses indicated that means of BMI differed significantly by whether they were theory-based and interventions of more than six months in duration (Table 2).

The updated first paragraph in Section 4 now reads as follows:

This review and meta-analysis synthesised the evidence of the efficacy of school-based interventions at changing obesity-related behaviours among primary-school children. Meta-analysis results indicate that school-based interventions had a small significant effect in BMI z-score compared with controls, but no significant effect on sedentary time, energy intake, fruit and vegetable intake and BMI (kg/m^2^). Subgroup analyses found studies lasting more than six months targeting a change in MVPA were more effective than studies that were of a shorter duration. In addition, we found significant interaction between theory-based interventions and interventions lasting more than six months and the effectiveness of changing BMI.

The updated second sentence in the fifth paragraph in Section 4 now reads as follows:

Over half of the included studies in this review did not specify the use of a behaviour change model or theory, and subgroup analyses inferred an effect on BMI z-score for interventions that were underpinned by behaviour change theory; however, although significant, the effect size was small.

The updated conclusion now reads as follows:

Although a small significant intervention effect was found between groups in BMI z-scores, overall the findings were inconsistent, and the heterogeneity observed across all outcomes was not explained by subgrouping. Furthermore, the meta-analyses of the included interventions to prevent childhood obesity were inconclusive regarding MVPA, SB, nutrition behaviour and for BMI kg/m^2^ compared with the control condition. This review highlights the need to better understand how implementation of multi-component interventions deals with various intervention targets—perhaps it is just too much for schools to take on. It is important that future research investigates whether consecutive rather than concurrent sequencing of delivery is better. To conclude, the chance of success may be greater in a single-component intervention that only targets one obesity-related behaviour. It is important policy makers continue to recognise the school setting as a vehicle for tackling childhood obesity.

The amended results do not significantly alter the paper’s overall conclusions or take-home messages, which underline the need to better understand how the implementation of multi-component school-based interventions deals with various intervention targets and that it is important that future research investigates whether consecutive rather than concurrent sequencing of delivery is better. The findings of the paper emphasise the importance that policymakers recognise the school setting as a vehicle for tackling childhood obesity.

The authors state that the scientific conclusions are unaffected. This correction was approved by the Academic Editor. The original publication has also been updated.
