# Supplementary material for: The Effectiveness of School-Based Interventions on Obesity-Related Behaviours in Primary School Children: A Systematic Review and Meta-Analysis of Randomised Controlled Trials
Source: Children (Basel). 2021 Jun 8;8(6):489. doi: 10.3390/children8060489 (PMC8228296; doi:10.3390/children8060489)
Supplement: Supplementary file 1 [file children-08-00489-s001.zip › Table S2 Search Strategy.pdf]

Additional File S2

Additional File S2: Search Strategy

MEDLINE

**1**	Child/
**2**	minors/
**3**	(child* OR young person OR young people OR minORs OR minOR).mp.
**4**	1 OR 2 OR 3
**5**	schools/
**6**	education/
**7**	students/
**8**	(school* OR educat* OR classroom OR student*).mp.
**9**	5 OR 6 OR 7 OR 8
**10**	exercise/
**11**	sports/
**12**	(exercise* OR sport* OR physical activit*).mp.
**13**	10 OR 11 OR 12
**14**	diet/
**15**	feeding behavior/
**16**	eating/
**17**	energy intake/
**18**	nutritional status/
**19**	(diet* OR feeding behavio?r OR eat* OR energy intake* OR nutritional status).mp.
**20**	sedentary lifestyle/
**21**	sedentary.mp.
**22**	20 OR 21
**23**	randomized controlled trials as Topic/
**24**	cross-over studies/
**25**	double-blind method/
**26**	(randomised OR randomized).mp.
**27**	23 OR 24 OR 25 OR 26
**28**	14 OR 15 OR 16 OR 17 OR 18 OR 19
**29**	13 OR 22 OR 28
**30**	4 AND 9 AND 27 AND 29
