# Supplementary material for: The Effectiveness of School-Based Interventions on Obesity-Related Behaviours in Primary School Children: A Systematic Review and Meta-Analysis of Randomised Controlled Trials
Source: Children (Basel). 2021 Jun 8;8(6):489. doi: 10.3390/children8060489 (PMC8228296; doi:10.3390/children8060489)
Supplement: Supplementary file 1 [file children-08-00489-s001.zip › Table S3 Characteristics of Included Studies.pdf]

Additional File S3

Description of the included trials (n = 48)

Author, year	Study design, Country	Aim/objective	Baseline characteristics	Intervention Description	Intervention outcomes: BMI	PA	SB	NB
Adab et al., 2018 [97]	Cluster RCT, UK	To assess the effectiveness of a school and family based healthy lifestyle programme in preventing childhood obesity.	**n =** 1392 (54 schools)<br>**Age**: 6.3 ± 0.3 yrs;<br>**Female (%)**: 48.9;<br>**Attrition rate**: 1 school (1.9%), 143 students (10.3%);<br>**Length of intervention:** 12 mons;<br>**Assessment:** Baseline, Post-IV, 3 months Post-IV, 18 Post-IV	**Underlying theory:** not mentioned;<br>**Intervention setting:** school and home;<br>**Content and frequency:** (1) 30 mins additional MVPA on each school day (2) Termly cooking workshops (3) A six-week programme to encourage healthy eating and increase PA (4) Information sheets signposting children and their families on ways to be active and PA opportunities in their local area;<br>**CON:** usual practice	BMI z-score<br><br>**Outcome Measure:** UK 1990 BMI reference curves for children)	PA EE (kJ/kg BW/day)<br>MVPA (min/day)<br><br>**Outcome Measure:** Actiheart	Sedentary time (min/day)<br><br>**Outcome Measure:** Actiheart	Energy intake (kJ/day)<br>Total fat (g/day)<br>Total sugar (g/day)<br>Total fibre (g/day)<br>Fruit and vegetable intake (g/day and portions)<br><br>**Outcome Measure:** Child and Diet Evaluation Tool (CADET)
Amini et al., 2016 [60]	Cluster RCT, Iran	To evaluate the effect of an intervention for reducing excess weight gain in primary school-age children.	**n =** 334 (12 schools)<br>**Age:** 8-12 yrs;<br>**Female (%)**: 49;<br>**Attrition rate**: N/A;<br>**Length of intervention:** 4.5 mons;<br>**Assessment:** Baseline, Post-IV	**Underlying theory:** not mentioned;<br>**Intervention setting:** school and home;<br>**Content and frequency:** (1) nutrition education for pupils, (2) lifestyle education for parents, (3) PA education for pupils (4) improvement of schools' canteens.<br>**CON:** no intervention	BMI z-score<br><br>**Outcome Measure:** AnthroPlus	METs<br><br>**Outcome Measure:** PA questionnaire	TV viewing/day (hr/day)<br>Working with computer/day (hr/day)<br><br>**Outcome Measure:** questionnaire	Energy intake (kcal/day)<br>Total fat (g/day)<br>Total protein (g/day)<br>Total carbohydrates (g/day)<br>Daily energy from fat (%)<br>Daily energy from carbohydrates (%)<br><br>**Outcome Measure:** 24-hr dietary recall
Anderson et al., 2016 [66]	Cluster RCT, UK	To investigate the long-term effectiveness of a school-based intervention to improve physical activity and diet in children.	**n =** 2221 (60 schools)<br>**Age**: 8–9 yrs;<br>**Female (%)**: 50;<br>**Attrition rate**: 54 (10%);<br>**Length of intervention:** 12 mons;<br>**Assessment:** Baseline, Post-IV, 12 mons Post-IV	**Underlying theory:** SCT;<br>**Intervention setting:** school and home;<br>**Content and frequency:** (1) Training for teachers, (2) 16 lesson-plans and teaching materials (3) 10 parental-child interaction homework activities (4) Information in the school newsletters about the importance of increasing PA, reducing SB & improving diet (5) information for parents on how to encourage their	BMI z-score<br><br>**Outcome Measure:** weight and height measured in classrooms (kg/m^2; treated in all analyses as an SD z-score)	MVPA (min/day)<br><br>**Outcome Measure:** ActiGraph GT3X+	Sedentary time (min/day)<br>Time spent screen viewing (min/day)<br>**Outcome Measure:** ActiGraph GT3X+ and self-reported questionnaire	Intake of fruit, vegetable, high-fat foods, snacks and high-energy drinks (number/day)<br><br>**Outcome Measure:** A Day in the Life self-reported questionnaire

				children to eat healthily and be active;<br>**CON:** usual practice				
Angelopoulos et al., 2009 [87]	Cluster RCT, Greece	To evaluate the effectiveness of this school-based intervention program, on obesity indices and blood pressure in primary school children.	**n =** 646 (26 schools)<br>**Age**: 10.3 ± 0.4 yrs;<br>**Female (%)**: 47.6;<br>**Attrition rate**: 0;<br>**Length of intervention:** 12 mons;<br>**Assessment:** Baseline, Post-IV	**Underlying theory:** Theory of Planned Behaviour;<br>**Intervention setting:** school and home;<br>**Content and frequency:** (1) student workbook and a teacher's manual was created & used in class (2) 2 x 45-min PE sessions per week delivered in the playground (3) increased accessibility to playgrounds and school yards (4) meetings to support parents (5) educational dietary activities<br>**CON:** no intervention	BMI (kg/m^2)<br>BMI z-score<br><br>**Outcome Measure:** Centres for Disease Control (CDC) 2000 Growth Charts.	MVPA (min/day)<br><br>**Outcome Measure:** Self-report questionnaire		Intake of fruit, vegetable, dairy, sweets and beverages, snack, grain, meat, fat and oil (portions/day)<br><br>**Outcome Measure:** 24-h recall
Bacardí-Gascon et al., 2012 [80]	Quasi Cluster RCT, Mexico	To assess the effect of a six-month intervention on the BMI, food consumption and PA of 2nd and 3rd grade elementary school children.	**n =** 532 (4 schools)<br>**Age**: 8.5 ± 0.7 yrs;<br>**Female (%)**: 49;<br>**Attrition rate**: 54 (11%);<br>**Length of intervention:** 6 mons;<br>**Assessment:** Baseline, Post-IV, 3 mons Post-IV, 7 mons Post-IV, 18 mons Post-IV.	**Underlying theory:** Bronfenbrenner's Ecological Model;<br>**Intervention setting:** school and home;<br>**Content and frequency:** (1) Three 60-minute sessions to discuss healthy lifestyles with school board & teachers (2) One 30-min interactive lesson was delivered by nutrition graduate students each week for 8 weeks (3) parents received a 60-minute session delivered by nutrition professionals each month for 4 months.<br>**CON:** after the initial six-month period they received the intervention.	BMI (kg/m^2)<br>BMI z-score<br><br>**Outcome Measure:** WHO 2007 LMS parameters	Outdoor play (h/day)<br>Physical Education (h/week)<br>Supervised sports or dancing (h/week)<br><br>**Outcome Measure:** Actigraph; INTA PA questionnaire; Subgroup analysis (n = 45)<br>.	Sitting (h/day)<br>TV watching (h/day)<br>Computer and video games (h/day)<br><br>**Outcome Measure:** Self-reported questionnaire	Intake of fruit, vegetable, dairy, sugar sweetened beverages, snack, soda, chocolate, and candy (portions/day)<br><br>**Outcome Measure:** Dietary registry and questionnaire on food inventory; (on a randomly selected weekday)
Bere et al., 2014 [103]	Cluster RCT, Norway	To assess if increased consumption of fruits and vegetables, due to free school fruit, have an impact on future weight status.	**n =** 1,950 (38 schools)<br>**Age**: 11.8 ± 0.7 yrs;<br>**Female (%)**: 49.5;<br>**Attrition rate**: 1,630 (83.6%);<br>**Length of intervention:** 9 mons;<br>**Assessment:** Baseline, Post-IV, 12 mons Post-IV,	**Underlying theory:** not reported;<br>**Intervention setting:** School;<br>**Content and frequency:** (1) Free school fruit for the school year<br>**CON:** not reported	BMI (kg/m^2)<br><br>**Outcome Measure:** Self-reported height and weight.			Intake of unhealthy snacks, fruit and vegetable (times/week)<br><br>**Outcome Measure:** 24-H recall & FFQ

			36 mons Post-IV, 18 mons Post-IV					
Brandstetter et al., 2012 [100]	Cluster RCT, Germany	To examine the effects of URMEL-ICE (Ulm Research on Metabolism, Exercise, and Lifestyle Intervention in Children), on children's BMI and other measures of fat mass.	**n =** 1119 (32 schools)<br>**Age**: 7.6 ± 0. yrs;<br>**Female (%)**: 46.5;<br>**Attrition rate**: not reported;<br>**Length of intervention:** 1 school year;<br>**Assessment:** Baseline, Post-IV.	**Underlying theory:** SCT;<br>**Intervention setting:** school and home;<br>**Content and frequency:** 29 teaching units designed to promote health-behaviour change in (1) sugar-sweetened beverages consumption (2) screen media (3) PA.<br>**CON:** not reported.	BMI (kg/m^2)<br>BMI z-score<br><br>**Outcome Measure:** age and sex-specific BMI charts for Germany	Outdoor play (times/week)<br>Participation in club sport and in other sport activity (times/week)<br>**Outcome Measure:** Parent questionnaire KiGGS survey	TV viewing (h/day)<br><br>**Outcome Measure:** Parent questionnaire KiGGS survey	Intake of sugar-sweetened beverages at home/school (consuming more or less)<br><br>**Outcome Measure:** Parent questionnaire KiGGS survey
Donnelly et al., 2009 [83]	Cluster RCT, USA	The aim of this study, Physical Activity Across the Curriculum (PAAC) was to promote PA and diminish increases in overweight and obesity in elementary school children.	**Subsample n =** 454 (24 schools),<br>**Age**: 8.3 yrs;<br>**Female (%)**: 46.5;<br>**Attrition rate**: 37 (2.5%);<br>**Length of intervention:** 36 mons;<br>**Assessment:** Baseline, Post-IV.	**Underlying theory:** not reported;<br>**Intervention setting:** School;<br>**Content and frequency:** (1)90 minutes/wk of MVPA active academic lessons delivered intermittently throughout the school day.<br>**CON:** regular classroom instruction without physically active lessons.	BMI (kg/m^2)<br>BMI z-score<br><br>**Outcome Measure:** BMI percentiles calculated using gender & age	MVPA (min/day)<br><br>**Outcome Measure:** ActiGraph, 7163, Pensacola, FL)		
Drummy et al., 2016 [61]	Cluster RCT, Northern Ireland	To examine the effect of a classroom-based activity break on accelerometer-determined MVPA and adiposity in primary school children.	**n =** 120 (14 schools)<br>**Age**: 9.5 yrs;<br>**Female (%)**: not specified;<br>**Attrition rate**: N/A;<br>**Length of intervention:** 3 mons;<br>**Assessment:** Baseline, Post-IV.	**Underlying theory:** not reported;<br>**Intervention setting:** School;<br>**Content and frequency:** (1) 5-min classroom-based activity break 3 times per day for 12 weeks.<br>**CON:** usual practice.	BMI (kg/m^2)<br><br>**Outcome Measure:** Height and weight	MVPA (min/day)<br><br>**Outcome Measure:** Actigraph GT1M		
Efstathiou et al., 2016 [81]	RCT, Greece	To measure classroom psychological climate & investigate its relationship to students' PA & sedentary life habits following an innovative education programme on nutrition and PA.	**n =** 1119 (32 schools)<br>**Age**: 7.6 ± 0. yrs;<br>**Female (%)**: not specified;<br>**Attrition rate**: N/A;<br>**Length of intervention:** 7 mons;<br>**Assessment:** Baseline, Post-IV.	**Underlying theory:** not reported;<br>**Intervention setting:** School;<br>**Content and frequency:** (1) *Intensive Intervention Group*: curriculum focused on theatrical with the participation of specialised personnel (nutritionists and life scientists) for 20 weekly class sessions (2) *Intervention Group:* nutrition education programme delivered over 20 weekly class sessions.<br>**CON:** did not participate in the nutrition education programme.		Outdoor PA (times/week)<br><br>**Outcome Measure:** Self-reported questionnaire	Screen time on weekdays (times/week)<br><br>**Outcome Measure:** Self-reported questionnaire	Not reported.<br><br>**Outcome Measure:** Self-reported questionnaire

Engelen et al., 2013 [67]	Cluster RCT, Australia.	To explore the effects of an innovative school-based intervention for increasing PA.	**n =** 226 (12 schools)<br>**Age**: 6.0 ± 0.6. yrs;<br>**Female (%)**: 46.2;<br>**Attrition rate**: N/A;<br>**Length of intervention:** 13 weeks;<br>**Assessment:** Baseline, Post-IV.	**Underlying theory:** not reported;<br>**Intervention setting:** School playground;<br>**Content and frequency:** (1) altering the school playground by introducing loose materials and (2) a teacher–parent intervention exploring perceptions of risk associated with children's free play;<br>**CON:** participated in standard break time.	BMI (kg/m^2)<br><br>**Outcome Measure:** Height and weight	Total accelerometer counts<br>MVPA (min/day)<br><br>**Outcome Measure:** ActiGraph GT3X+	Sedentary time (min/24 hrs)<br><br>**Outcome Measure:** ActiGraph GT3X+	
Fairclough et al., 2013 [68]	Cluster RCT, UK.	This evaluation investigated the effectiveness of the Children's Health, Activity and Nutrition: Get Educated! (CHANGE!) Project, an intervention to promote healthy weight using an educational focus on PA and healthy eating.	**n =** 318 (12 schools)<br>**Age**: 10.6 ± 0.3 yrs;<br>**Female (%)**: not specified;<br>**Attrition rate**: 112 (35.2%);<br>**Length of intervention:** 20 weeks;<br>**Assessment:** Baseline, Post-IV, 10 weeks Post-IV.	**Underlying theory:** SCT;<br>**Intervention setting:** School and home;<br>**Content and frequency:** (1) teacher-led curriculum (20 weekly lessons, 60 min each), (2) learning resources, (3) homework tasks;<br>**CON:** received normal instruction.	BMI (kg/m^2)<br>BMI z-score<br><br>**Outcome Measure:** BMI percentiles calculated using gender & age	LPA, MPA, VPA (min/day)<br><br>**Outcome Measure:** ActiGraph GT1M	Sedentary time (min/24 hrs)<br><br>**Outcome Measure:** ActiGraph GT1M	Consumption of breakfast cereals, breads, meats, dairy products (times/day)<br><br>**Outcome Measure:** 24-hour recall food intake questionnaire
Farmer et al., 2017 [84]	Cluster RCT, New Zealand.	The aim of the PLAY study was to determine whether providing greater opportunities for risk and challenge in primary schools increased PA and reduced relative body weight over the long term.	**n =** 840 (16 schools)<br>**Age**: 8.0 ± 1.1 yrs;<br>**Female (%)**: 51;<br>**Attrition rate**: 210 (25%);<br>**Length of intervention:** 12 mons;<br>**Assessment:** Baseline, Post-IV, 12 mons Post-IV.	**Underlying theory:** not reported;<br>**Intervention setting:** School;<br>**Content and frequency:** redesigned play environment (1) promoting greater challenges, 2) adding more dynamic equipment, 3) relaxing the rules.<br>**CON:** schools were asked to not change anything in their school play spaces.	BMI (kg/m^2)<br>BMI z-score<br><br>**Outcome Measure:** BMI percentiles calculated using gender & age	Estimate of overall PA (counts/min)<br>MVPA (min/day)<br><br>**Outcome Measure:** ActiGraph GT3X		
Ford et al., 2013 [98]	RCT, UK.	To establish whether an accumulated brisk walking programme, performed during the school day, is effective in changing body composition	**n =** 174 (2 schools)<br>**subsample n** = 121<br>**Age**: 5-11 yrs;<br>**Female (%)**: 50.1;<br>**Attrition rate**: 22 (12.7%);<br>**Length of intervention:** 15 weeks;<br>**Assessment:** Baseline, Post-IV.	**Underlying theory:** not reported;<br>**Intervention setting:** School;<br>**Content and frequency:** (1) brisk walk around the school grounds for 15 min in the morning and afternoon, at least three times a week, for a total of 90 min per week;<br>**CON:** usual practice.	BMI (kg/m^2)<br>BMI z-score<br><br>**Outcome Measure:** BMI percentiles calculated using gender & age	Total accelerometer counts<br><br>**Outcome Measure:** MTI Accelerometers		Energy intake (kcal/day)<br><br>**Outcome Measure:** Parental 3-day dietary recall

Habib-Mourad et al., 2020 [77]	RCT, Lebanon.	To describe the effectiveness of a school-based intervention when delivered by a non-nutrition specialist (trained schoolteachers) as compared to an expert in nutrition.	**n =** 2276 (52 schools)<br>**Age**: 9-11 yrs;<br>**Female (%)**: not given;<br>**Attrition rate**:<br>128 (5.6%);<br>**Length of intervention:**<br>12 mons;<br>**Assessment:** Baseline, Post-IV.	**Underlying theory:** SCT;<br>**Intervention setting:**<br>School and home;<br>**Content and frequency:**<br>(1) 12 classroom sessions incorporated into the school curriculum, 2) family module consisting of information , 3) relaxing the rules.<br>**CON:** usual curriculum		Sessions of PE (times/week)<br>Outdoor play (times/week) After school PA (times/week)<br><br>**Outcome Measure:**<br>Self-reported questionnaire	Daily breakfast intake<br>Snack intake (times/day)<br>Eating in front of the TV (times/week)<br><br>**Outcome Measure:**<br>Self-reported questionnaire
Howe et al., 2011 [59]	RCT, USA.	To determine if a 10-month after-school PA intervention could prevent deleterious changes in body composition and cardiovascular fitness in young black boys.	**n =** 106 (5 schools)<br>**Age**: 8-12 yrs;<br>**Female (%)**: 0;<br>**Attrition rate**:<br>22 (12.7%);<br>**Length of intervention:**<br>10 mons;<br>**Assessment:** Baseline, Post-IV.	**Underlying theory:** not reported;<br>**Intervention setting:**<br>School;<br>**Content and frequency:**<br>set afterschool and included (1)30 minutes of homework time during which the boys were provided with a healthy snack (2) followed by 80 minutes of PA;<br>**CON: no intervention and were not allowed to stay for the after-school intervention.**	BMI (kg/m^2)<br>**Outcome Measure:**<br>Height and weight	MVPA, LPA, MPA, VPA (min/day)<br><br>**Outcome Measure:**<br>seven-day PA recall	
Kain et al., 2014 [92]	Cluster RCT, Chile.	To evaluate the effectiveness of a 12-month multicomponent obesity prevention intervention.	**n =** 1949 (9 schools)<br>**Age**: 6.6 ± 1.1 yrs;<br>**Female (%)**: 46.6;<br>**Attrition rate**:<br>475 (24.4%);<br>**Length of intervention:**<br>12 mons;<br>**Assessment:** Baseline, Post-IV.	**Underlying theory:** not reported;<br>**Intervention setting:**<br>school;<br>**Content and frequency:**<br>(1) classroom education on nutrition, 2) improve the quality of PE classes;<br>**CON:** usual practice.	BMI (kg/m^2)<br>BMI z-score<br><br>**Outcome Measure:**<br>WHO reference	MVA in PE classes (min/day)<br><br>**Outcome Measure:**<br>pedometers (New Lifestyle 1000)	
Khan et al., 2014 [99]	RCT, USA.	To investigate the effect of a 9-month PA intervention on cardiorespiratory fitness and adiposity among prepubertal children.	**n =** 220 (7 schools)<br>**dietary subsample n =** 135<br>**Age**: 8.8 ± 0.6 yrs;<br>**Female (%)**:46.8;<br>**Attrition rate**:<br>27 (12.3%);<br>**Length of intervention:**<br>9 mons;<br>**Assessment:** Baseline, Post-IV.	**Underlying theory:** not reported;<br>**Intervention setting:**<br>school;<br>**Content and frequency:**<br>2-hour intervention (5 days/week for 9 mons), (1) each session began with 20-25 mins PA station, (2) healthy snack provided during the 15-minute educational component;<br>**CON:** regular after-school routine.	BMI (kg/m^2)<br>BMI z-score<br><br>**Outcome Measure:**<br>Height and weight		Total energy intake (kcal/day) Total fat, protein, carbohydrate intake (%/kcal).<br><br>**Outcome Measure:**<br>24-hour food recall;

Kobel et al., 2014 [63]	Cluster RCT, Germany	To evaluate children's behaviours in respect of increased PA, a decrease in screen media use (SMU), more regular breakfast, and a reduction of the consumption of soft drinks.	**n =** 1943 (154 schools)<br>**Age**: 7.1 ± 0.6 yrs;<br>**Female (%)**: 48.8;<br>**Attrition rate**: 207 (10.7%);<br>**Length of intervention:** 12 mons;<br>**Assessment:** Baseline, Post-IV.	**Underlying theory:** SCT and Bronfenbrenner's social ecological model;<br>**Intervention setting:** School and home;<br>**Content and frequency:** based on teaching materials (1) offering action alternatives for recreational activities (without screen media), (2) PA, and a healthy diet (focusing on breakfast and soft drinks) which are integrated into the primary school curriculum<br>**CON:** usual practice.	BMI z-score<br><br>**Outcome Measure:** calculated as weight divided by height squared and converted to BMI percentiles (BMIPCT) using German reference data to define their weight status.	MVPA (min/day)<br><br>**Outcome Measure:** parental questionnaire<br><br>.	Screen time (min/day)<br><br>**Outcome Measure:** parental questionnaire<br><br>.	Intake of soft drink consumption (serving/day)<br><br>**Outcome Measure:** parental questionnaire<br><br>.
Kobel et al., 2017 [64]	Cluster RCT, Germany	To evaluate children's behaviours from a migration background in respect of increased PA, a decrease in screen media use (SMU), more regular breakfast, and a reduction of the consumption of soft drinks.	**n =** 525 (154 schools)<br>**Age**: 7.1 ± 0.7 yrs;<br>**Female (%)**: 51.4;<br>**Attrition rate**: N/A;<br>**Length of intervention:** 12 mons;<br>**Assessment:** Baseline, Post-IV.	**Underlying theory:** SCT and Bronfenbrenner's social ecological model;<br>**Intervention setting:** School and home;<br>**Content and frequency:** based on teaching materials (1) offering action alternatives for recreational activities (without screen media), (2) PA, and a healthy diet (focusing on breakfast and soft drinks) which are integrated into the primary school curriculum<br>**CON:** usual practice.	BMI z-score<br><br>**Outcome Measure:** calculated as weight divided by height squared and converted to BMI percentiles using German reference data to define their weight status.	MVPA (min/day)<br><br>**Outcome Measure:** parental questionnaire<br>.	Screen time (min/day)<br><br>**Outcome Measure:** parental questionnaire<br><br>.	Intake of soft drink consumption (serving/day)<br><br>**Outcome Measure:** parental questionnaire<br><br>.
Kocken et al., 2016 [91]	RCT, Netherland s	To evaluate the effectiveness of the "Extra Fit!" education program in promoting healthy diet and PA to prevent and reduce overweightness among primary school children.	**n =** 1112 (45 schools)<br>**Age**: 7.1 yrs;<br>**Female (%)**: 51.7;<br>**Attrition rate**: N/A;<br>**Length of intervention:** 2 school yrs;<br>**Assessment:** Baseline, 7-mons Post baseline, Post-IV, 6 mons Post-IV.	**Underlying theory:** The Theory of Planned Behaviour;<br>**Intervention setting:** School and home;<br>**Content and frequency:** based on classroom and physical education activities, including practical assignments, group discussions and lessons to change (1) dietary intake, (2) PA and (3) SB.<br>**CON:** usual practice.	BMI z-score<br><br>**Outcome Measure:** Weight and height	MVPA (min/day)<br><br>**Outcome Measure:** Actigraph and questionnaire for children	Sedentary and screen time (min/day)<br><br>**Outcome Measure:** questionnaire for children	Energy intake (kcal/day), fat, protein, sugar sweetened beverages, other beverages, carbohydrates, fruit and vegetable (g/day)<br><br>**Outcome Measure:** 24-h recall for children.
Lau et al., 2016 [85]	RCT, China	To determine the effect of a school based AVG	**n =** 80 (1 school)<br>**Age**: 9.2 ± 0.5 yrs;	**Underlying theory:** not reported;<br>**Intervention setting:** School;	BMI (kg/m^2)	MVPA (min/day)		

		intervention on Chinese children's aerobic fitness, PA level, and PA-related psychological correlates.	**Female (%)**: 31.3; **Attrition rate**: 0; **Length of intervention:** 12 weeks; **Assessment:** Baseline, Post-IV.	**Content and frequency:** (1) played active videogames, Xbox 360, twice per week during after-school hours, each for 60 minutes; **CON:** usual practice.	**Outcome Measure:** Weight and height	**Outcome Measure:** ActiGraph GT3X+		
Li et al., 2019 [69]	Cluster RCT, China	To evaluate the clinical- and cost-effectiveness of the Chinese Primary School Children Physical Activity and Dietary Behaviour Changes Intervention developed using the UK Medical Research Council complex intervention framework to prevent obesity in Chinese primary-school–aged children.	**n =** 1641 (40 schools) **Age**: 9.2 ± 0.5yrs; **Female (%)**: 54.5; **Attrition rate**: 55 (3.4%); **Length of intervention:** 12 mons; **Assessment:** Baseline, Post-IV.	**Underlying theory:** A theoretical pathway informed by a mixed-methods study; **Intervention setting:** School and home; **Content and frequency:** Targeting PA and NB (1) Interactive education workshops for carers with summarising leaflet, (2) adjustments to school lunches, (3) taster session to teach fun & active PA games and PA homework assignment. **CON:** usual practice.	BMI z-score<br><br>**Outcome Measure:** Weight and height	(1) MVPA (min/day) (2) Parental reported MVPA (min/day) (3) Proportion of children achieving ≥60 minutes MVPA/day<br><br>**Outcome Measure:** (1) GENEActiv Original, Activinsights Ltd, (2) Adapted Godin Leisure-Time Exercise Questionnaire (3) questionnaire	(1) Sedentary time (min/day) (2) Parent reported sedentary behaviours (mins/24 hours) (3) Screen time (h/day)<br><br>**Outcome Measure:** (1) GENEActiv Original, Activinsights Ltd, (2) Adapted Godin Leisure-Time Exercise Questionnaire (3) questionnaire	Weekly average servings of unhealthy snacks and sugar added drinks Proportion of children consuming ≥5 portions of fruit and vegetables daily Daily average portions of fruit and vegetables<br><br>**Outcome Measure:** food frequency questionnaire;
Liu et al., 2019 [102]	Cluster RCT, China	A comprehensive approach involving both environmental and individual strategies offers opportunities to strengthen school-based interventions for childhood obesity.	**n =** 1641 (12 schools) **Age**: 7-11 yrs; **Female (%)**:48.3; **Attrition rate**: 50 (2.6%); **Length of intervention:** 12 mons; **Assessment:** Baseline, Post-IV.	**Underlying theory:** the analysis grid for environments linked to obesity (ANGELO); **Intervention setting:** School and home; **Content and frequency:** (1) school policies: certain food and beverages and electronic devices not permitted in the school, (2) health education activities, (3) 3 x 45-minute PE classes per week; **CON:** usual practice.	BMI (kg/m^2) BMI z-score<br><br>**Outcome Measure:** Weight and height	PA (min/day)<br><br>**Outcome Measure:** Self-administered 7-day PA questionnaire<br><br>.	Screen time (h/day)<br><br>**Outcome Measure:** Self-administered questionnaire<br><br>.	Intake of meat, sugar-sweetened beverages, fruit and vegetable (portion/day)<br><br>**Outcome Measure:** Self-administered questionnaire based on the validated Block Kids Food Screener
Llargues et al., 2011 [58]	Cluster RCT, Spain	To evaluate the effect of an intervention that modified food and PA habits on the progression of BMI in a population of school children using Research, Vision,	**n =** 598 (16 schools) **Age**: 6.0 ± 0.3 yrs; **Female (%)**:46; **Attrition rate**: 89 (14.9%) for anthropometric data; **Length of intervention:** 24 mons; **Assessment:** Baseline, Post-IV.	**Underlying theory:** not reported; **Intervention setting:** School and home; **Content and frequency:** (1) activities about healthy food habits and PA in classroom 3hr/week; (2) hand out healthy recipes for the families; (3) give out materials on healthy foods and	BMI (kg/m^2)<br><br>**Outcome Measure:** Weight and height	PA at weekends Walking before/during/ after school<br><br>**Outcome Measure:** Kreece Plus test	TV video or DVD (h/day)<br><br>**Outcome Measure:** Kreece Plus test<br><br>.	Foods consumed at breakfast. Daily intake of fruit or juice, dairy, raw or cooked vegetables, fish, fast food, legumes, sweets and olive oil

		Action and Change (IVAC) methodology.		about promoting healthy PA; (4) provide equipment for games; **CON:** usual practice.		.		**Outcome Measure:** Kreece Plus test
Llaurado et al., 2014 [104]	RCT, Spain	To assess the reproducibility of an educational intervention EdAl-2 (Educació en Alimentació) programme in 'Terres de l'Ebre' over 22 months, to improve lifestyles, including diet and PA.	**n =** 916 (16 schools) **Age**: 8.04 ± 0.6; **Female (%)**:47.7; **Attrition rate**: 411 (17.5%); **Length of intervention:** 22 mons; **Assessment:** Baseline, Post-IV.	**Underlying theory:** not reported; **Intervention setting:** School and home; **Content and frequency:** (1) eight lifestyle topics incorporated within 12 activities which were disseminated over 12 sessions (1 h/activity/ session), and prepared, standardised and implemented as four activities per school academic year in the classroom; **CON:** usual practice.	BMI (kg/m^2) BMI z-score **Outcome Measure:** Weight and height	PA at weekends Walking before/during/after school **Outcome Measure:** Kreece Plus test (parental questionnaire)	TV video or DVD (h/day) **Outcome Measure:** Kreece Plus test (parental questionnaire)	Foods consumed at breakfast. Daily intake of fruit or juice, dairy, raw or cooked vegetables, fish, fast food, legumes, sweets and olive oil; **Outcome Measure:** Kreece Plus test (parental questionnaire)
Lloyd et al., 2018 [101]	Cluster RCT, UK	To establish whether a school-based intervention for children aged 9–10 years would prevent excessive weight gain after 24 months.	**n =** 1371 (32 schools) **Age**: 9.8 ± 0.3yrs; **Female (%)**:51.5; **Attrition rate**: 32 (2%) of children opted out by their parents or carers; **Length of intervention:** 9 mons; **Assessment:** Baseline, 9 mons Post-IV, 15 mons Post-IV.	**Underlying theory:** SCT; **Intervention setting:** School and home; **Content and frequency:** (1) creating a supportive context: professional sports people and dancers run practical workshops, (2) intensive healthy lifestyle week, (3) 10 mins personal goal setting discussion, (4) reinforce all the messages through an assembly and lesson; **CON:** usual practice.	BMI (kg/m^2) BMI z-score **Outcome Measure:** Weight and height .	MVPA, LPA, MPA, VPA (min/day) **Outcome Measure:** GENEActiv	Sedentary(min/day) **Outcome Measure:** GENEActiv	Energy-dense snack intake and healthy snack intake (portions/day) **Outcome Measure:** Food Intake Questionnaire
Lynch et al., 2016 [56]	Cluster RCT, USA	To evaluate the impact of the Let's Go! 5-2-1-0 paediatric obesity intervention program in an elementary school setting.	**n =** 51 (1 schools) **Age**: 8.0 yrs; **Female (%)**:54.9; **Attrition rate**: 20 (39.2%); **Length of intervention:** 4 mons; **Assessment:** Baseline, Post-IV	**Underlying theory:** not reported; **Intervention setting:** School; **Content and frequency:** (1) 8 sessions anchored around the 5-2-1-0 curriculum (key daily messages of the program are for children to eat at least 5 servings of fruits and vegetables, limit recreational screen time to 2 or fewer hours, participate in at least 1 hour of PA, and to ingest 0 sugar-containing drinks) **CON:** usual practice.	BMI (kg/m^2) **Outcome Measure:** Weight and height	Step count (step/day) Improved PA (score out of 10) **Outcome Measure:** Omron HJ-321 pedometer and caregiver survey	Screen time (h/day) **Outcome Measure:** Caregiver survey	Beverage intake (portion/day) Improved eating behaviour (score out of 10) **Outcome Measure:** Caregiver survey
Madsen et al., 2015 [70]	Cluster RCT, USA	To assess the impact of Energy Balance for Kids with Play, a	**n =** 879 (6 schools) **Age**: 9.8 ± 0.3yrs;	**Underlying theory:** not reported; **Intervention setting:** School;	BMI (kg/m^2)	MVPA (min/day)	School day sedentary(min/day)	Fruit and vegetable consumption (cups)

		school-based intervention developed by the Academy of Nutrition and Dietetics Foundation and Playworks, on students' PA, dietary habits and knowledge, and weight status.	**Female (%)**:53;<br>**Attrition rate**: 212 (24%);<br>**Length of intervention:** 24 mons;<br>**Assessment:** Baseline, Post-IV.	**Content and frequency:** (1) 12-week nutrition and energy balance education curriculum, (2) implement classroom wellness policies, (3) Packaging equipment for the school kitchen, (4) PA sessions and 4 afterschool sports leagues each year;<br>**CON:** usual practice.	**Outcome Measure:** Weight and height	**Outcome Measure:** Actigraph GT1M or GT3X	**Outcome Measure:** Actigraph GT1M or GT3X	**Outcome Measure:** Digital images and survey questions adapted from the School PA and Nutrition Questionnaire and the Child Food Consumption Questionnaire
Marcus *et al.* (2009) [57]	Cluster RCT, Sweden	To assess whether a school-based prevention programme focused on reduced unhealthy eating and increased PA during school time over a 4-year period could reduce the prevalence of overweight and obesity among 6 to 10-year-old children.	**n =** 3135 (10 schools)<br>**Age**: 9.5 ± 0.7yrs;<br>**Female (%)**:49;<br>**Attrition rate**: 69 (14.1%);<br>**Length of intervention:** 4 years;<br>**Assessment:** Baseline, Post-IV.	**Underlying theory:** not reported;<br>**Intervention setting:** School and home;<br>**Content and frequency:** change the school environment by (1) increase amount of PA by 30 min per child per day, (2) increased number of vegetables served at school lunch, (3) sweets, sweet buns and ice cream were eliminated at festivities;<br>**CON:** usual practice.	BMI (kg/m^2)<br><br>**Outcome Measure:** Weight and height	Daily PA (min/day)<br><br>**Outcome Measure:** Actiwatch		Intake of meat, sweet drinks, bread, dairy products, fruit and vegetable (portion/day)<br><br>**Outcome Measure:** Parental questionnaire
Martinez-Vizcaino et al., 2014 [71]	Cluster RCT, Spain	To assess the impact of a standardized physical activity program on adiposity and cardiometabolic risk factors in schoolchildren.	**n =** 1070 (20 schools)<br>**Age**: 9.5 ± 0.7yrs;<br>**Female (%)**: 49;<br>**Attrition rate**: 69 (14.1%);<br>**Length of intervention:** 9 mons;<br>**Assessment:** Baseline, Post-IV.	**Underlying theory:** Socio-ecological model;<br>**Intervention setting:** School and home;<br>**Content and frequency:** (1) play-based and non-competitive activities and is conducted for two 90-minute sessions on weekdays and one 150-minute session on Saturday mornings;<br>**CON:** usual practice. The standard physical education curriculum (2 h per week of PA at low-to-moderate intensity) continued to be taught in both the control and intervention schools.	BMI (kg/m^2)<br><br>**Outcome Measure:** Weight and height<br>.	Daily PA (min/day)<br><br>**Outcome Measure:** ActiGraph MTI/CSA	Sedentary (min/day)<br><br>**Outcome Measure:** ActiGraph MTI/CS	
Nathan et al., 2020 [72]	Cluster RCT, Australia	To assess the impact of a multi strategy intervention designed to improve teachers' implementation of a school PA policy on student PA levels.	**n =** 2148 (12 schools)<br>**Age**: 9.5 ± 2.0 yrs;<br>**Female (%)**: 49;<br>**Attrition rate**: 646 (30%);<br>**Length of intervention:** 9 mons;<br>**Assessment:** Baseline, Post-IV.	**Underlying theory:** theoretical domains framework;<br>**Intervention setting:** School;<br>**Content and frequency:** (1) 5 face-to-face workshop delivered by support officers, (2) various printed and	.	Total MVPA (min/day)<br><br>**Outcome Measure:** ActiGraph GT3X+ accelerometer	Sedentary (min/day)<br><br>**Outcome Measure:** ActiGraph GT3X+ accelerometer	

				electronic instructional materials to assist<br>scheduling and implementation of PA, (3) support officers;<br>**CON:** usual PA practice.				
Nickel et al., 2020 [96]	Cluster RCT, Canada	To determine whether improvements documented in the Healthy Buddies intervention were experienced by all children taking part in the intervention and across several different social determinants.	**n =** 687 (20 schools)<br>**Age**: 6-12 yrs;<br>**Female (%)**: 48;<br>**Attrition rate**: 104 (15%);<br>**Length of intervention:** 9 mons;<br>**Assessment:** Baseline, Post-IV.	**Underlying theory:** not reported;<br>**Intervention setting:** School;<br>**Content and frequency:** (1) 21 healthy living lessons taught weekly in the classroom, (2) two 30-min structured PA sessions per week;<br>**CON:** usual practice.	BMI z-score<br><br>**Outcome Measure:** Centres for Disease Control and Prevention growth charts			Knowledge and attitudes to healthy eating (3-point scale for frequency)<br><br>**Outcome Measure:** Self-administered questionnaire
O'Leary et al., 2019 [86]	RCT, Ireland	To assess the impact of Project Spraoi: a school-based PA and nutrition intervention that reached 473 primary school children and 43 school staff in Cork (Ireland).	**n =** 473 (4 schools)<br>**Age**: 6 yrs and 10 yrs<br>**Female (%)**: not reported;<br>**Attrition rate** N/A;<br>**Length of intervention:** 2 yrs;<br>**Assessment:** Baseline, Post-IV.	**Underlying theory:** RE-AIM (Reach, Efficacy, Adoption, Implementation, Maintenance) theoretical framework;<br>**Intervention setting:** School and home;<br>**Content and frequency:** (1) promotion of 20 min 'huff and puff' (MVPA) each day, (2) improvement of nutritional habits and knowledge through targeted class-based activities, (3) promotion of increased habitual PA, (4) reduced sedentary time;<br>**CON:** usual practice.	BMI (kg/m^2)<br><br>**Outcome Measure:** Weight and height	MVPA (min/day)<br><br>**Outcome Measure:** ActiGraph GT3X and wGT3X+		Knowledge and attitudes to healthy eating<br><br>**Outcome Measure:** nutritional knowledge and attitudes questionnaires
Rausch Herscovici et al., 2013 [94]	RCT, Argentina	To evaluate the impact of a school-based obesity prevention program that seeks to change food intake among students at schools in Rosario, Argentina.	**n =** 387 (6 schools)<br>**Age**: 9.5 ± 0.7 yrs;<br>**Female (%)**:48;<br>**Attrition rate**: 18 (5%);<br>**Length of intervention:** 6 mons;<br>**Assessment:** Baseline, Post-IV.	**Underlying theory:** not reported;<br>**Intervention setting:** School and home;<br>**Content and frequency:** (1) four workshops (healthy eating, body in motion, healthy body &for parent/caregiver), plus modifications to the school cafeteria menu;<br>**CON:** usual practice.	BMI (kg/m^2)<br>BMI z-score<br><br>**Outcome Measure:** Weight and height			Intake of fruit and vegetable, skimmed milk, cereal, juice, sweet and chocolate and sweetened beverage<br><br>**Outcome Measure:** Weekly Food Frequency Questionnaire
Resaland et al., 2016 [73]	Cluster RCT, Norway	To investigate the effect of a seven-month, school-based cluster-randomized	**n =** 1129 (57 schools)<br>**Age**: 10.2 ± 0.3 yrs;	**Underlying theory:** Socio-ecological framework - socio-ecological model, Harter's Competence Motivation Theory,	BMI (kg/m^2)	Total PA, LPA, MVPA (min/day)	Sedentary (min/day)	

		controlled trial on academic performance in 10-year-old children.	**Female (%)**:48; **Attrition rate**: 40 (4%); **Length of intervention:** 7 mons; **Assessment:** Baseline, Post-IV.	Achievement goal theory and Ryan & Deci's self-determination theory; **Intervention setting:** School and home; **Content and frequency:** (1) physically active lessons, (2) PA breaks (5 min/day) implemented in the classroom during academic lessons; and (3) PA homework (10 min/day; **CON:** usual practice, including usual amounts of PA/PE, being approximately 135 min/week.	**Outcome Measure:** Weight and height	**Outcome Measure:** ActiGraph GT3X+	**Outcome Measure:** ActiGraph GT3X+	
Rosario et al., 2017 [88]	RCT, Portugal	To evaluate the impact of an intervention program, taught by trained teachers, on foods and nutrients components of the Diet Quality Index–International among children in Grades 1 to 4.	**n =** 464 (7 schools) **Age**: 8.3 ± 1.2 yrs; **Female (%)**: 51.5; **Attrition rate:** 170 (36.6); **Length of intervention:** 4 mons; **Assessment:** Baseline, Post-IV.	**Underlying theory:** Health promotion model and SCT; **Intervention setting:** School; **Content and frequency:** (1) Teachers had 12 sessions of 3 hours each with the study researchers **CON:** usual practice.	BMI (kg/m^2)<br>BMI z-score<br>**Outcome Measure:** Weight and height	PA index (%/day)<br>**Outcome Measure:** Questionnaire (4-point scale)		Diet Quality Index–International<br>**Outcome Measure:** 24-h dietary recall
Sacchetti et al., 2013 [75]	RCT, Italy	To assess whether a school-based physical education intervention was effective in improving physical abilities and influencing daily PA habits in primary school children.	**n =** 497 (26 schools) **Age**: 8-9 yrs; **Female (%)**: 51.5; **Attrition rate:** 69 (13.9%); **Length of intervention:** 2 yrs; **Assessment:** Baseline, Post-IV.	**Underlying theory:** not reported; **Intervention setting:** School; **Content and frequency:** (1) daily 45 min of MVPA in multi-environments (schoolyard, classroom, gym); **CON:** usual physical education programme.	BMI (kg/m^2)<br>**Outcome Measure:** Weight and height	Daily PA (%/day)<br>**Outcome Measure:** Physical Activity Questionnaire for children	Sedentary (%/day)<br>**Outcome Measure:** Physical Activity Questionnaire for children	
Santina et al., 2020 [78]	Cluster RCT, Lebanon	To evaluate the efficacy of a theory- and evidence-based intervention for promoting PA among children by changing PA-related psychosocial variables and reducing obesity and screen time outside school hours.	**n =** 384 (2 schools) **Age**: 10.6 ± 0.6 yrs; **Female (%)**: 45.7; **Attrition rate:** 10 (3%); **Length of intervention:** 14 weeks; **Assessment:** Baseline, Post-IV.	**Underlying theory:** Social identity theory; **Intervention setting:** School; **Content and frequency:** (1)) 10-min daily structured MVPA, (2) 20-min daily structured and unstructured MVPA during recess, (3) letters to parents to increase their knowledge about PA, (4) 4-day training of educational staff, (5) daily monitoring of children's participation in PA, (6) additional PA equipment, (7) school events promoting PA, (8) earning	BMI (kg/m^2)<br>**Outcome Measure:** Weight and height	PAQ-C score<br>**Outcome Measure:** Physical Activity Questionnaire for Older Children (PAQ-C)	Screen time (h/day)<br>**Outcome Measure:** Physical Activity Questionnaire for children	

				activities of PA delivered in classroom;<br>**CON:** received the standard school curriculum.			
Scherr et al., 2017 [93]	Cluster RCT, USA.	To investigate the effectiveness of a multicomponent, school-based nutrition intervention, the Shaping Healthy Choices Program (SHCP), to improve children's dietary behaviours and prevent childhood obesity	**n =** 443 (4 schools)<br>**Age**: 9.6 yrs;<br>**Female (%)**: 49.7;<br>**Attrition rate:** not given;<br>**Length of intervention:** 9 mons;<br>**Assessment:** Baseline, Post-IV.	**Underlying theory:**<br>SCT, Social Ecological Model;<br>**Intervention setting:**<br>School;<br>**Content and frequency:**<br>(1) nutrition education and promotion (15 classroom lessons & 19 take-home activities), (2) family and community partnership, (3) supporting regional agriculture, (4) foods available on the school campus, (5) school wellness and policies;<br>**CON:** The nutrition educator spent time in the control classrooms to match the number of hours spent in the intervention classrooms content was unrelated to nutrition, health, and science.	BMI (kg/m^2)<br>BMI z-score<br><br>**Outcome Measure:**<br>Weight and height		Nutrition knowledge score<br>Fruit and vegetable intake (servings/day)<br>**Outcome Measure:**<br>Food frequency questionnaire<br><br>.
Seljebotn et al., 2019 [74]	Cluster RCT, Norway	To report effects of the Active School program on objectively measured PA level and aerobic fitness. In addition, the PA level during the 45-minute physically active lessons was compared to ordinary classroom lessons and physical education lessons.	**n =** 447 (4 schools)<br>**Age**: 9.6 yrs;<br>**Female (%)**: 49;<br>**Attrition rate:** not given;<br>**Length of intervention:** 10 mons;<br>**Assessment:** Baseline, Post-IV.	**Underlying theory:** not reported;<br>**Intervention setting:**<br>School and home;<br>**Content and frequency:**<br>(1) PA increase by 190min/week, (2) PA homework and (3) physically active lessons;<br>**CON:** usual practice.	BMI (kg/m^2)<br>BMI z-score<br><br>**Outcome Measure:**<br>Weight and height	MVPA, LPA, MPA, VPA (min/day)<br><br>**Outcome Measure:**<br>ActiGraph GT1M/GT3X/GT3 X+	Sedentary (min/day)<br><br>**Outcome Measure:**<br>ActiGraph GT1M/GT3X/GT3 X+
Siegrist et al., 2013 [89]	RCT, Germany	To evaluate a simple and ubiquitously applicable school-based educational program to increase PA, fitness, and lifestyle awareness and to improve health obesity measures.	**n =** 724 (8 schools)<br>**Age**: 8.4 ± 0.7 yrs;<br>**Female (%)**:<br>**Attrition rate:** 102 (14%);<br>**Length of intervention:** 12 mons;<br>**Assessment:** Baseline, Post-IV.	**Underlying theory:** not reported;<br>**Intervention setting:**<br>School and home;<br>**Content and frequency:**<br>(1) JuvenTUM intervention monthly lessons lasting 45 min with three parts: a warm-up of 10 min with running, playing running games at high intensity, 30 min exercises to improve body awareness and self-esteem with	BMI (kg/m^2)<br><br>**Outcome Measure:**<br>Weight and height	Active days/week (≥60 min)<br><br>**Outcome Measure:**<br>Questionnaire	

				conversation in class about health-related topics, and 5 min relaxation exercises;<br>**CON:** usual practice.				
Tarro et al., 2014 [76]	Cluster RCT, Spain	To implement a program by university students acting as “health promoting agents” and to evaluate the effects on obesity prevalence of the primary-school-based program that promotes healthy lifestyle.	**n =** 2350 (38 schools)<br>**Age**: 8.4 ± 0.6 yrs;<br>**Female (%)**: 49.9;<br>**Attrition rate:**<br>411 (17.5%);<br>**Length of intervention:**<br>28 mons;<br>**Assessment:** Baseline, Post-IV.	**Underlying theory:** not reported;<br>**Intervention setting:**<br>School and home;<br>**Content and frequency:**<br>(1) eight lifestyle topics incorporated within 12 activities which were disseminated over 12 sessions (1 h/activity/ session), and prepared, standardised and implemented as four activities per school academic year in the classroom;<br>**CON:** usual practice.	BMI (kg/m^2)<br>BMI z-score<br><br>**Outcome Measure:**<br>Weight and height<br>.	PA at weekends<br>Walking before/during/after school<br><br>**Outcome Measure:**<br>Kreece Plus test (parental questionnaire)<br><br>.	TV video or DVD (h/day)<br><br>**Outcome Measure:**<br>Kreece Plus test (parental questionnaire)<br><br>.	Breakfast consumption, has a dairy product for breakfast, eats cereals or derivative for breakfast, manufactured pastries for breakfast<br>Intake of fruit or juice daily, dairy product, raw or cooked vegetables a day, fish consumption, fast food, legumes, sweets and olive oil;<br>**Outcome Measure:**<br>Kreece Plus test (parental questionnaire)
Telford et al., 201 [62]	Cluster RCT, Australia	To investigate the effect of a 4-year specialist-taught Physical Education program on PA among primary school children.	**n =** 853 (29 schools)<br>**Age**: 8.1 ± 0.4;<br>**Female (%)**: 49%;<br>**Attrition rate**:<br>313 (37%);<br>**Length of intervention:**<br>4 yrs;<br>**Assessment:**<br>Baseline, 1 yr, 2 yrs, 3 yrs ,Post-IV.	**Underlying theory:** not reported;<br>**Intervention setting:**<br>School;<br>**Content and frequency:**<br>(1) 2 × 45 min PE lessons per week from specialist-trained PE teachers;<br>**CON:** usual practice.	BMI (kg/m^2)<br><br>**Outcome Measure:**<br>Weight and height	1. PA during PE classes<br>2. Total PA (steps/day)<br>3. MVPA (min/day)<br><br>**Outcome Measure:**<br>1. System for Observing Fitness Instruction Time<br>2. Pedometers<br>3. Actigraph GT1M	Sedentary time (min/day)<br><br>**Outcome Measure:**<br>Pedometer<br><br>.	
Viggiano et al., 2018 [90]	Cluster RCT, Italy	To investigate whether the board game Kaledo improves nutritional knowledge and helps to promote a healthy lifestyle in children.	**n =** 1313 (10 schools)<br>**Age**: 7-11 yrs;<br>**Female (%)**: not reported;<br>**Attrition rate**:<br>857 (65.3%);<br>**Length of intervention:**<br>20 weeks;	**Underlying theory:** not reported;<br>**Intervention setting:**<br>School;<br>**Content and frequency:**<br>(1) participated in one play session (15–30 min) with the board game Kaledo, every week;	BMI (kg/m^2)<br>BMI z-score<br><br>**Outcome Measure:**<br>Weight and height	PA frequency and duration (min/day)<br><br>**Outcome Measure:**<br>WHO Health Behaviour in		Intake of healthy and junk food (portions/day).<br><br>**Outcome Measure:**<br>7-day food diary<br>.

			**Assessment:**<br>Baseline, 8 mons, 10 mons Post-IV.	**CON:** did not play with Kaledo.		School-aged Children (HBSC) PA questionnaire		
Wells et al., 2014 [82]	Cluster RCT, USA	The study examines effects of a school garden intervention on elementary school children's PA	**n =** 264 (12 schools)<br>**Age**: 10.2 ± 0.5 yrs;<br>**Female (%)**: 47.7;<br>**Attrition rate**: 30 (11%);<br>**Length of intervention:** 12 mons;<br>**Assessment:**<br>Baseline, Post-IV.	**Underlying theory:** not reported;<br>**Intervention setting:**<br>School garden;<br>**Content and frequency:**<br>(1) garden bed raised, (2) 2 lessons, (3) resources for the school, (4) garden implementation guide;<br>**CON:** received gardens at the end of the study.		MVPA, MPA, LPA, VPA (min/day)<br>PA activity<br><br>**Outcome Measure:**<br>(1) GEMS Activity Questionnaire<br>(2) Actigraph GT3X+/GT1M<br>(3) PA Research & Assessment tool for Garden Observation (PARAGON)	Sedentary time (min/day)<br><br>**Outcome Measure:**<br>(1) GEMS Activity Questionnaire<br>(2) Actigraph GT3X+/GT1M<br>(3) PA Research & Assessment tool for Garden Observation (PARAGON)	
Williamson et al., 2012 [79]	Cluster RCT, USA	The study was designed to test the efficacy of a Primary Prevention program (PP) and a combination of PP and a Secondary Prevention (SP) program in comparison to a Control group for prevention of weight/fat gain in the entire sample and overweight children	**n =** 2060 (17 schools)<br>**Age**: 10.5 ± 1.2 yrs;<br>**Female (%)**: 58.4;<br>**Attrition rate**:<br>363 (17.6%);<br>**Length of intervention:** 18 mons;<br>**Assessment:** Baseline, Post-IV, 12 mons Post-IV.	**Underlying theory:**<br>The Theory of Triadic Influence, the Comprehensive School Health Program Model;<br>**Intervention setting:**<br>School and home;<br>**Content and frequency:**<br>three prevention arms: (1) Primary Prevention an environmental modification program, (2) Primary + Secondary Prevention, the environmental program with an added classroom and internet education component;<br>**CON:** has none of the prevention components that are hypothesized to yield weight gain prevention. This control arm can be viewed as a nonspecific control condition.	BMI (kg/m^2)<br>BMI z-score<br><br>**Outcome Measure:**<br>Weight and height	Total PA (min/day)<br><br>**Outcome Measure:**<br>Self-Administered Physical Activity Checklist (SAPAC)	Sedentary time (min/day)<br><br>**Outcome Measure:**<br>Self-Administered Physical Activity Checklist (SAPAC)	Total energy intake, total fat, protein, carbohydrate intake (kcal/day).<br><br>**Outcome Measure:**<br>(Sony DCR-TRV22) digital video camera to measure food selection and food intake

Xu et al., 2015 [95]	Cluster RCT, China	To assess the effectiveness of a school-based multi-component lifestyle childhood obesity prevention program	**n =** 1108 (8 schools)<br>**Age**: 10.2 ± 0.5 yrs;<br>**Female (%)**: 47.7;<br>**Attrition rate**: 74 (6.3%);<br>**Length of intervention:** 8 mons;<br>**Assessment:** Baseline, Post-IV.	**Underlying theory:**<br>The Theory of Triadic Influence, the Comprehensive School Health Program Model;<br>**Intervention setting:**<br>School and home;<br>**Content and frequency:**<br>classroom curriculum (1) 30-min lesson/mon, (2) school environment support, (3) family involvement, (4) fun programs/events;<br>**CON:** usual health and physical education curriculum.	BMI (kg/m^2)<br><br>**Outcome Measure:**<br>Weight and height	PA patterns<br>Commuting mode<br><br>**Outcome Measure:**<br>validated Chinese version of the International Physical Activity Questionnaire (CHN-IPAQ)	Patterns of TV viewing<br><br>**Outcome Measure:**<br>validated Chinese version of the International Physical Activity Questionnaire (CHN-IPAQ)	Consumption of breakfast cereals, snack, fast food, soft drink, meats, dairy products (times/day)<br><br>**Outcome Measure:**<br>FFQ

Abbreviations: RCT: randomized controlled trial; UK: United Kingdom; CON: control group; yrs: years; mons: months; BMI: body mass index; PA: physical activity; SB: sedentary behaviour; EE: energy expenditure; NB: nutrition behaviour; PE: physical education; wk.:week; d: day; hr: hour; min: minute; MVPA: moderate-to-vigorous physical activity; LPA: low-intensity physical activity; MPA: moderate-intensity physical activity; VPA: vigorous physical activity; Post-IV: post-intervention; kcal: calories; SCT: social cognitive theory; FFQ: food frequency questionnaire.
